# Classification of anisotropic Triebel-Lizorkin spaces

**DOI:** 10.1007/s00208-023-02690-y

**Published:** 2023-08-04

**Authors:** Sarah Koppensteiner, Jordy Timo van Velthoven, Felix Voigtlaender

**Affiliations:** 1 Faculty of Mathematics, University of Vienna, Oskar-Morgenstern-Platz 1, 1090 Vienna, Austria; 2 Delft University of Technology, Mekelweg 4, Building 36, 2628 CD Delft, The Netherlands; 3 Mathematical Institute for Machine Learning and Data Science (MIDS), Catholic University of Eichstätt-Ingolstadt (KU), Auf der Schanz 49, 85049 Ingolstadt, Germany

**Keywords:** 42B25, 42B35, 46E35

## Abstract

This paper provides a characterization of expansive matrices $A \in \textrm{GL}(d, {\mathbb {R}})$ generating the same anisotropic homogeneous Triebel–Lizorkin space $\dot{\textbf{F}}^{\alpha }_{p,q}(A)$ for $\alpha \in {\mathbb {R}}$ and $p,q \in (0,\infty ]$. It is shown that $\dot{\textbf{F}}^{\alpha }_{p,q}(A) = \dot{\textbf{F}}^{\alpha }_{p,q}(B)$ if and only if the homogeneous quasi-norms $\rho _A, \rho _B$ associated to the matrices *A*, *B* are equivalent, except for the case $\dot{\textbf{F}}^0_{p, 2} = L^p$ with $p \in (1,\infty )$. The obtained results complement and extend the classification of anisotropic Hardy spaces $H^p(A) = \dot{\textbf{F}}^{0}_{p,2}(A)$, $p \in (0,1]$, in Bownik (Mem Am Math Soc 164(781):vi+122, 2003).

## Introduction

Let $A \in \textrm{GL}(d, {\mathbb {R}})$ be an expansive matrix and consider an analyzing vector $\varphi \in {\mathcal {S}}({\mathbb {R}}^d)$ for *A*, that is, a Schwartz function $\varphi : {\mathbb {R}}^d \rightarrow {\mathbb {C}}$ with Fourier transform $\widehat{\varphi } \in C_c^{\infty } ({\mathbb {R}}^d {\setminus } \{0\})$ satisfying

$$\begin{aligned} \sup _{i \in {\mathbb {Z}}} |\widehat{\varphi } ((A^*)^i \xi )| > 0 \quad \text {for all} \quad \xi \in {\mathbb {R}}^d {\setminus } \{0\}, \end{aligned}$$

where $A^*$ denotes the transpose of *A*. Denote its $L^1$-normalized dilation by $\varphi _i:= |\det A|^i \varphi (A^i \cdot )$ for $i \in {\mathbb {Z}}$. For $\alpha \in {\mathbb {R}}$ and $p, q \in (0,\infty ]$, the associated anisotropic homogeneous Triebel–Lizorkin space $\dot{\textbf{F}}^{\alpha }_{p,q}(A)$ on ${\mathbb {R}}^d$ is defined to consist of all tempered distributions $f \in {\mathcal {S}}' ({\mathbb {R}}^d)$ (modulo polynomials) with finite quasi-norm $\Vert f \Vert _{\dot{\textbf{F}}^{\alpha }_{p,q}(A)}$, defined by

$$\begin{aligned} \Vert f \Vert _{\dot{\textbf{F}}^{\alpha }_{p,q}(A)} = \bigg \Vert \bigg ( \sum _{i \in {\mathbb {Z}}} (|\det A|^{\alpha i} \, |f *\varphi _i |)^q \bigg )^{1/q} \bigg \Vert _{L^p}, \quad p \in (0,\infty ), \end{aligned}$$

with the usual modifications for $q = \infty$, and

$$\begin{aligned} \Vert f \Vert _{\dot{\textbf{F}}^{\alpha }_{\infty ,q}(A)} = \sup _{\ell \in {\mathbb {Z}}, k \in {\mathbb {Z}}^d} \bigg ( \frac{1}{|\det A|^{\ell }} \int _{A^{\ell }([0,1]^d + k)} \sum _{i = -\ell }^{\infty } (|\det A|^{\alpha i} \, |(f *\varphi _i)(x)|)^q \; d x \bigg )^{1/q}, \end{aligned}$$

and $$\Vert f \Vert _{\dot{\textbf{F}}^{\alpha }_{\infty ,\infty }(A)} = \sup _{i \in {\mathbb {Z}}} |\det A|^{\alpha i} \Vert f *\varphi _i \Vert _{L^{\infty }}$$.

For the scalar dilation matrix $A = 2 \cdot I_d$, the spaces $\dot{\textbf{F}}^{\alpha }_{p,q}(A)$ defined above coincide with the usual homogeneous Triebel–Lizorkin spaces on ${\mathbb {R}}^d$ as studied in, e.g., [15, 16, 24]. For this particular case, the Triebel–Lizorkin spaces provide a unifying scale of function spaces that encompasses, among others, the Lebesgue, Sobolev, Hardy and BMO spaces. The anisotropic Triebel–Lizorkin spaces $\dot{\textbf{F}}^{\alpha }_{p,q}(A)$ associated to a general expansive matrix *A* were first introduced in [6] and further studied in, e.g., [1, 4, 5, 8, 18–20]. These anisotropic spaces are useful for the analysis of mixed homogeneity properties of functions and operators as the dilation structure allows different directions to be scaled by different dilation factors. Among others, the anisotropic Triebel–Lizorkin spaces include Lebesgue spaces and various anisotropic/parabolic versions of Hardy and BMO spaces as studied in, e.g., [2, 7, 9–11, 14]. See these papers (and the references therein) for further motivation for considering anisotropic function spaces.

In the present paper, the main objective is to characterize when two expansive matrices induce the same anisotropic Triebel–Lizorkin space. The problem of classifying anisotropic Triebel–Lizorkin spaces can be traced back to [22], where the question of dependence of the anisotropic Triebel–Lizorkin sequence spaces on diagonal dilation matrices is raised as [22, Conjecture 11]; see also [23, Section 5.3]. For the case of anisotropic Hardy spaces $H^p(A)$ ($= \dot{\textbf{F}}^0_{p,2} (A)$) with $p \in (0,1]$, a full solution to this problem for general expansive matrices *A* has been obtained in [2]. Explicitly, it is shown in [2, Section 10] that $H^p(A) = H^p(B)$ for some (equivalently, all) $p \in (0,1]$ if and only if two homogeneous quasi-norms $\rho _A, \rho _B: {\mathbb {R}}^d \rightarrow [0,\infty )$ associated to the expansive matrices *A*, *B* are equivalent, in the usual sense of quasi-norms. See also [7] for a slightly corrected version and [13] for an extension of the classification result of [2] to Hardy spaces with variable anisotropy. Analogous to these results on Hardy spaces, a classification of anisotropic Besov spaces [3] has more recently been obtained in [12]. The aim of this paper is to provide a complementary characterization for the scale of Triebel–Lizorkin spaces.

### Main results

The first result obtained in this paper gives a sufficient condition for two expansive matrices generating the same anisotropic Triebel–Lizorkin space. Here, as well as below, two expansive matrices *A* and *B* are called *equivalent* if they have equivalent homogeneous quasi-norms; see Sects. 2.1 and 2.2 for precise definitions.

#### Theorem 1.1

If $A, B \in \textrm{GL}(d, {\mathbb {R}})$ are equivalent expansive matrices, then $\dot{\textbf{F}}^{\alpha }_{p,q}(A) = \dot{\textbf{F}}^{\alpha }_{p,q}(B)$ for all $\alpha \in {\mathbb {R}}$ and $p,q \in (0,\infty ]$.

The following rigidity theorem provides a converse to Theorem 1.1.

#### Theorem 1.2

Let $A, B \in \textrm{GL}(d, {\mathbb {R}})$ be expansive, $\alpha , \beta \in {\mathbb {R}}$ and $p_1, p_2, q_1, q_2 \in (0, \infty ]$.

If $\dot{\textbf{F}}^{\alpha }_{p_1,q_1}(A) = \dot{\textbf{F}}^{\beta }_{p_2,q_2}(B)$, then $(p_1, q_1, \alpha ) = (p_2, q_2, \beta )$. Furthermore, at least one of the following conditions hold:

(i) *A* and *B* are equivalent, or
(ii) $$\alpha = \beta = 0$$, $$p_1 = p_2 \in (1, \infty )$$ and $$q_1 = q_2 = 2$$.

Theorem 1.2 shows, in particular, that equivalence of two expansive matrices is necessary for the coincidence of the associated spaces, unless $\alpha = 0$, $p \in (1, \infty )$ and $q = 2$. That this conclusion might fail for the space $\dot{\textbf{F}}^0_{p, 2} (A)$ with $p \in (1,\infty )$ is easily explained, namely $\dot{\textbf{F}}^0_{p, 2} (A)$ can be canonically identified with the Lebesgue space $L^p$ for $p\in (1,\infty )$, see, e.g., [2, 4].

A combination of Theorems 1.1 and 1.2 provides a full characterization of two expansive matrices inducing the same anisotropic Triebel–Lizorkin space. This characterization extends the classification of anisotropic Hardy spaces [2] to the full scale of Triebel–Lizorkin spaces, while complementing the classification of anisotropic Besov spaces [12] with a counterpart for Triebel–Lizorkin spaces.

In effect, the aforementioned classification theorems translate the problem of comparing function spaces into the comparison of homogeneous quasi-norms. For this latter problem, explicit and verifiable criteria in terms of spectral properties of the involved dilation matrices can be given, see, e.g., [2, Section 10], [12, Section 7] and [7, Section 4].

As an illustration of Theorem 1.2, we note that a matrix $B \in \textrm{GL}(d, {\mathbb {R}})$ is equivalent to the scalar dilation $A = 2 \cdot I_d$ if and only if *B* is diagonalizable over ${\mathbb {C}}$ with all eigenvalues equal in absolute value, see, e.g., [2, Example, p.7]. Combined with Theorem 1.2, this shows that for matrices *B* that are not of this special form,

$$\begin{aligned} \dot{\textbf{F}}^{\alpha }_{p,q}(A) \ne \dot{\textbf{F}}^{\alpha }_{p,q}(B), \end{aligned}$$

unless $\alpha = 0$, $p \in (1,\infty )$ and $q = 2$. In particular, the (homogeneous) Sobolev spaces $L^p_{\alpha }$ ($= \dot{\textbf{F}}^{\alpha }_{p, 2} (A)$) with $1< p < \infty$ and $\alpha \ne 0$ do *not* coincide with $\dot{\textbf{F}}^{\alpha }_{p, 2} (B)$ for non-diagonalizable matrices *B*.

Lastly, let us mention an application of Theorem 1.1. In [18, 19], we proved *continuous* maximal characterizations of anisotropic Triebel–Lizorkin spaces $\dot{\textbf{F}}^{\alpha }_{p,q}(A)$ and obtained new results on their molecular decomposition. These results were obtained under the additional assumption that the expansive matrix *A* is exponential, in the sense that $A = \exp (C)$ for some matrix $C \in {\mathbb {R}}^{d \times d}$. Theorem 1.1 implies that this additional assumption does not restrict the scale of anisotropic Triebel–Lizorkin spaces. Indeed, since there always exists an expansive *and* exponential matrix *B* that is equivalent to the given expansive matrix *A* (cf. [12, Section 7]), it follows by Theorem 1.1 that $\dot{\textbf{F}}^{\alpha }_{p,q}(A) = \dot{\textbf{F}}^{\alpha }_{p,q}(B)$ for all $\alpha \in {\mathbb {R}}$ and $p,q \in (0,\infty ]$.

### Methods

An essential ingredient in our proof of Theorems 1.1 and 1.2 is a simple characterization of the equivalence of two expansive matrices *A* and *B* in terms of properties of the associated covers $\bigl ( (A^*)^i Q\bigr )_{i \in {\mathbb {Z}}}$ and $\bigl ( (B^*)^j P\bigr )_{j \in {\mathbb {Z}}}$ of ${\mathbb {R}}^d {\setminus } \{0\}$, where $P, Q \subseteq {\mathbb {R}}^d {\setminus } \{0\}$ are suitable relatively compact sets; see [12, Lemma 6.2] and Sect. 2.3. Explicitly, this criterion asserts that two expansive matrices *A*, *B* are equivalent if and only if the associated homogeneous covers $\bigl ( (A^*)^i Q\bigr )_{i \in {\mathbb {Z}}}$ and $\bigl ( (B^*)^j P\bigr )_{j \in {\mathbb {Z}}}$ satisfy

1.1 $$\begin{aligned} \sup _{i \in {\mathbb {Z}}} \big | \big \{ j \in {\mathbb {Z}} : (A^*)^i Q \cap (B^*)^j P \ne \varnothing \big \} \big | + \sup _{j \in {\mathbb {Z}}} \big | \big \{ i \in {\mathbb {Z}} : (A^*)^i Q \cap (B^*)^jP \ne \varnothing \big \} \big | < \infty . \end{aligned}$$

The formulation (1.1) of the equivalence of matrices *A* and *B* is what is actually used in the proofs of our main results, as we expand upon next.

#### Sufficient conditions

In the proof of Theorem 1.1, the criterion (1.1) is used to control the overlap of the Fourier supports of the *A*-dilates and *B*-dilates of the analyzing vectors $\varphi$ and $\psi$, respectively, that are used to define the spaces $\dot{\textbf{F}}^{\alpha }_{p,q}(A)$ and $\dot{\textbf{F}}^{\alpha }_{p,q}(B)$. Combined with our maximal characterizations of Triebel–Lizorkin spaces obtained in [18, 19], this allows to conclude that the analyzing vectors $\varphi$ and $\psi$ for *A* respectively *B* define the same space $\dot{\textbf{F}}^{\alpha }_{p,q}(A) = \dot{\textbf{F}}^{\alpha }_{p,q}(B)$.

#### Necessary conditions

In the proof of Theorem 1.2, we show the asserted equivalence of two matrices *A* and *B* by showing that the criterion (1.1) holds. For this, we first carefully construct auxiliary functions in $\dot{\textbf{F}}^{\alpha }_{p,q}(A) = \dot{\textbf{F}}^{\alpha }_{p,q}(B)$ whose Fourier supports are contained in finitely many of the sets $(A^*)^{i_k} Q$ and $(B^*)^{j_k} P$, where $i_k, j_k \in {\mathbb {Z}}$, of appropriate homogeneous covers $\bigl ( (A^*)^i Q\bigr )_{i \in {\mathbb {Z}}}$ and $\bigl ( (B^*)^j P\bigr )_{j \in {\mathbb {Z}}}$. Then, using adequate estimates of the norms of these auxiliary functions (see Sect. 5.2), it is shown directly that (1.1) must hold for the case $\alpha \ne 0$, in which case *A* and *B* must be equivalent. The proof strategy for the case $\alpha = 0$ is similar, but requires some additional arguments and tools. For $p < \infty$, it is shown using the Khintchine inequality that necessarily $q = 2$ whenever *A* and *B* are not equivalent. For $p = \infty$, we use dual norm characterizations of Triebel–Lizorkin norms to conclude that *A* and *B* must be equivalent.

As mentioned above, the used criterion (1.1) for equivalent matrices stems from [12], where it was used for the purpose of classifying anisotropic Besov spaces. For the actual comparison of function spaces, the approach of [12] consists of showing that an anisotropic Besov space can be identified with a (Besov-type) decomposition space [26], which allows to apply the embedding theory [26] developed by the third named author. In contrast, the Triebel–Lizorkin spaces considered in this paper cannot be directly treated in the framework [26]; in particular, our main theorems cannot be easily deduced from [26]. Some of our arguments for proving Theorem 1.2 are, however, inspired by ideas used in [26], most notably the use of the Khintchine inequality. Nevertheless, all of our calculations and estimates differ non-trivially from corresponding arguments in [26] as the latter concerns Besov-type norms, which are technically easier to deal with than the Triebel–Lizorkin norms considered in this paper.

### Organization

The overall structure of this paper is as follows: Sect. 2 collects various notions and results related to expansive matrices and associated homogeneous covers. The essential background on anisotropic Triebel–Lizorkin spaces is contained in Sect. 3. Theorem 1.1 is proven in Sect. 4, whereas Sect. 5 provides the proof of Theorem 1.2. Lastly, some technical auxiliary results are postponed to two appendices.

### Notation

For a measurable set $\Omega \subseteq {\mathbb {R}}^d$, we denote its Lebesgue measure by $\textrm{m}(\Omega )$ and the indicator function of $\Omega$ by $\mathbbm {1}_{\Omega }$. The notation $| \cdot |: {\mathbb {R}}^d \rightarrow [0, \infty )$ is used for the Euclidean norm. The open Euclidean ball of radius $r > 0$ and center $x \in {\mathbb {R}}^d$ is denoted by $B_r (x)$. The closure of a set $\Omega \subseteq {\mathbb {R}}^d$ will be denoted by $\overline{\Omega }$.

The Schwartz space on ${\mathbb {R}}^d$ is denoted by ${\mathcal {S}} ({\mathbb {R}}^d)$ and ${\mathcal {S}}' ({\mathbb {R}}^d)$ denotes its dual, the space of tempered distributions. For $f \in {\mathcal {S}}'({\mathbb {R}}^d)$ and $g \in {\mathcal {S}}({\mathbb {R}}^d)$, we define $\langle f, g \rangle := f(\overline{g})$, so that the dual pairing $\langle \cdot , \cdot \rangle$ is sesquilinear, in agreement with the inner product on $L^2({\mathbb {R}}^d)$. The subspace of ${\mathcal {S}}({\mathbb {R}}^d)$ consisting of functions with all moments vanishing (i.e., $\int x^\alpha \, f(x) \, dx = 0$ for all $\alpha \in {\mathbb {N}}_0^d$) is denoted by ${\mathcal {S}}_0 ({\mathbb {R}}^d)$. The dual space ${\mathcal {S}}'_0 ({\mathbb {R}}^d)$ is often identified with the quotient ${\mathcal {S}}' /{\mathcal {P}}$ of ${\mathcal {S}}'({\mathbb {R}}^d)$ and the space of polynomials ${\mathcal {P}}({\mathbb {R}}^d)$. Lastly, the space of smooth compactly supported functions on an open set $U \subseteq {\mathbb {R}}^d$ is as usual denoted by $C^{\infty }_c (U)$.

For a function $f: {\mathbb {R}}^d \rightarrow {\mathbb {C}}$, its translation $T_y f$ and modulation $M_y f$ by $y \in {\mathbb {R}}^d$ are defined by $T_y f = f(\cdot - y)$ and $M_y f = e^{2\pi i y \cdot } f$, respectively. The Fourier transform of $f \in L^1 ({\mathbb {R}}^d)$ is normalized as $\widehat{f} (\xi ) = \int _{{\mathbb {R}}^d} f(x) e^{- 2 \pi i \xi \cdot x} \; dx$ for $\xi \in {\mathbb {R}}^d$, where $\xi \cdot x = \sum _{j=1}^d \xi _j x_j$. The notation ${\mathcal {F}} f:= \widehat{f}$ is also sometimes used.

For two functions $f, g: X \rightarrow [0,\infty )$ on a set *X*, we write $f \lesssim g$ whenever there exists $C > 0$ such that $f(x) \le C g (x)$ for all $x \in X$. We simply use the notation $f \asymp g$ whenever $f \lesssim g$ and $g \lesssim f$. We also write $A \lesssim B$ for the inequality $A \le C B$, where $C > 0$ is constant independent of *A* and *B*. In case the implicit constant in $\lesssim$ depends on a quantity $\alpha$, we also sometimes write $\lesssim _\alpha$.

## Expansive matrices and homogeneous covers

This section collects background on expansive matrices and homogeneous quasi-norms. A standard reference for most of the presented material is [2].

### Expansive matrices

A matrix $A \in \textrm{GL}(d, {\mathbb {R}})$ is said to be *expansive* if $|\lambda | > 1$ for all $\lambda \in \sigma (A)$, where $\sigma (A) \subseteq {\mathbb {C}}$ denotes the spectrum of *A*. Throughout, we let $\lambda _-$ and $\lambda _+$ denote numbers such that $1< \lambda _- < \min _{\lambda \in \sigma (A)} |\lambda |$ and $\lambda _+ > \max _{\lambda \in \sigma (A)} |\lambda |$, and define $\zeta _+:= \ln \lambda _+ / \ln |\det A|$ and $\zeta _-:= \ln \lambda _- / \ln |\det A|$.

A set $\Omega \subseteq {\mathbb {R}}^d$ is an *ellipsoid* if $\Omega = \{ x \in {\mathbb {R}}^d: |P x| < 1 \}$ for some $P \in \textrm{GL}(d, {\mathbb {R}})$. Given any expansive matrix *A*, there exists an ellipsoid $\Omega _A$ and $r > 1$ such that

2.1 $$\begin{aligned} \Omega _A \subseteq r \Omega _A \subseteq A \Omega _A, \end{aligned}$$

and $\textrm{m} \left( {\Omega _A}\right) = 1$, see, e.g., [2, Lemma 2.2]. The choice of an ellipsoid satisfying (2.1) is not unique. Throughout, given an expansive matrix *A*, we will fix one choice of ellipsoid $\Omega _A$ associated to *A*.

### Homogeneous quasi-norms

Let $A \in \textrm{GL}(d, {\mathbb {R}})$ be an expansive matrix. A *homogeneous quasi-norm* associated with *A* is a measurable function $\rho : {\mathbb {R}}^d \rightarrow [0,\infty )$ satisfying the three properties:

1. $\rho (x) = 0$ if and only if $x = 0$;
2. $$\rho (A x) = |\det A| \rho (x)$$ for all $$x \in {\mathbb {R}}^d$$;
3. there exists $C > 0$ such that $\rho (x+y) \le C(\rho (x) + \rho (y))$ for all $x, y \in {\mathbb {R}}^d$.

By [2, Lemma 2.4], any two homogeneous quasi-norms $\rho _1, \rho _2$ associated to a fixed expansive matrix *A* are equivalent, in the sense that there exists $C > 0$ such that

2.2 $$\begin{aligned} \frac{1}{C} \rho _1 (x) \le \rho _2 (x) \le C \rho _1 (x) \end{aligned}$$

for all $$x \in {\mathbb {R}}^d$$.

In the sequel, we will primarily work with the so-called *step homogeneous quasi-norm* $\rho _A$ associated to *A*, defined as

$$\begin{aligned} \rho _A (x) = {\left\{ \begin{array}{ll} |\det A|^i, &{}\quad \text {if} \quad x \in A^{i+1} \Omega _A {\setminus } A^i \Omega _A, \\ 0, &{}\quad \text {if} \quad x = 0, \end{array}\right. } \end{aligned}$$

where $\Omega _A$ is the fixed expansive ellipsoid (2.1); see [2, Definition 2.5]. This quasi-norm is comparable to the Euclidean norm, in the sense that there exists $C \ge 1$ such that, for all $x \in {\mathbb {R}}^d$,

2.3 $$\begin{aligned} \frac{1}{C} [\rho _A (x)]^{\zeta _-}&\le | x | \le C [\rho _A(x)]^{\zeta _+}, \quad \text {if }\rho _A(x) \ge 1, \nonumber \\ \frac{1}{C} [\rho _A (x)]^{\zeta _+}&\le | x | \le C [\rho _A(x)]^{\zeta _-}, \quad \text {if }\rho _A(x) \le 1, \end{aligned}$$

see, e.g., [2, Lemma 3.2].

Two expansive matrices $A, B \in \textrm{GL}(d, {\mathbb {R}})$ are called *equivalent* if the associated step homogeneous quasi-norms $\rho _{A}$ and $\rho _{B}$ are equivalent. Note that, by Eq. (2.2), two expansive matrices are equivalent if and only if all of their associated quasi-norms are equivalent.

The following characterization is [2, Lemma 10.2].

#### Lemma 2.1

([2]) Let $A,B \in \textrm{GL}(d, {\mathbb {R}})$ be expansive. Then *A* and *B* are equivalent if and only if

$$\begin{aligned} \sup _{k \in {\mathbb {Z}}} \big \Vert A^{-k} B^{\lfloor c k \rfloor } \big \Vert < \infty , \end{aligned}$$

where $$c = c(A, B):= \ln |\det A| / \ln |\det B|$$.

As a corollary of the previous lemma (see also [12, Remark 4.9]), we see that equivalence of expansive matrices is preserved under taking transposes.

#### Corollary 2.2

Two expansive matrices *A* and *B* are equivalent if and only if $A^*$ and $B^*$ are equivalent.

### Homogeneous covers

Let $A \in \textrm{GL}(d, {\mathbb {R}})$ be expansive and let $Q \subseteq {\mathbb {R}}^d$ be open such that $\overline{Q}$ is compact in ${\mathbb {R}}^d {\setminus } \{0\}$. A cover $(A^i Q)_{i \in {\mathbb {Z}}}$ of ${\mathbb {R}}^d {\setminus } \{0\}$ is called a *homogeneous cover induced by* *A*. Given two homogeneous covers $(A^i Q)_{i \in {\mathbb {Z}}}$ and $(B^j P)_{j \in {\mathbb {Z}}}$ induced by $A, B \in \textrm{GL}(d, {\mathbb {R}})$, we define

2.4 $$\begin{aligned} J_i := \big \{ k \in {\mathbb {Z}} : A^i Q \cap B^k P \ne \varnothing \big \} \quad \text {and} \quad I_j := \big \{ k \in {\mathbb {Z}} : A^k Q \cap B^j P \ne \varnothing \big \} \end{aligned}$$

for fixed $$i, j \in {\mathbb {Z}}$$.

The index sets defined in Eq. (2.4) can be used for characterizing the equivalence of two expansive matrices as the following lemma shows. See [12, Lemma 6.2] for a proof.

#### Lemma 2.3

([12]) Let $A, B \in \textrm{GL}(d, {\mathbb {R}})$ be expansive and let $(A^i Q)_{i \in {\mathbb {Z}}}$ and $(B^jP)_{j \in {\mathbb {Z}}}$ be associated induced covers of ${\mathbb {R}}^d {\setminus } \{0\}$. Then the step homogeneous quasi-norms $\rho _A$ and $\rho _B$ are equivalent if and only if

$$\begin{aligned} \sup _{i \in {\mathbb {Z}}} |J_i | +\sup _{j \in {\mathbb {Z}}} |I_j| < \infty . \end{aligned}$$

In addition to Lemma 2.3, we will also make use of more refined estimates on the cardinalities of the index sets defined in Eq. (2.4). We provide the required estimates in the following two lemmata. The provided proofs follow arguments in the proof of Lemma 2.3 (cf. [12, Lemma 6.2]) closely, but are included here for completeness.

#### Lemma 2.4

Let $A, B \in \textrm{GL}(d, {\mathbb {R}})$ be two equivalent expansive matrices and $Q, P \subseteq {\mathbb {R}}^d$ open such that $\overline{Q}, \overline{P}$ are compact in ${\mathbb {R}}^d {\setminus } \{0\}$. Then there exists $C > 0$ such that

2.5 $$\begin{aligned} \frac{1}{C} |\det B|^j \le |\det A|^i \le C |\det B|^j \end{aligned}$$

whenever $i,j \in {\mathbb {Z}}$ are such that $A^i Q \cap B^j P \ne \varnothing$.

#### Proof

If $A^i Q \cap B^j P \ne \varnothing$, then there exists $x_0 \in Q \cap A^{-i}B^j P$. Hence, by homogeneity of $\rho _A, \rho _B$ and the assumption of their equivalence, it follows that

$$\begin{aligned} |\det B|^j \rho _B(B^{-j} A^i x_0) = \rho _B(A^i x_0) \ge \frac{1}{C} \rho _A(A^i x_0) = \frac{|\det A|^i}{C} \rho _A(x_0). \end{aligned}$$

Since $B^{-j} A^i x_0 \in P$, this yields

$$\begin{aligned} |\det A|^i \le C \frac{\max _{x \in \overline{P}}\{\rho _B(x)\}}{\min _{x \in \overline{Q}}\{\rho _A(x)\}} \, |\det B|^j, \end{aligned}$$

where $\max _{x \in \overline{P}}\{\rho _B(x)\} / \min _{x \in \overline{Q}}\{\rho _A(x)\}$ is finite by Eq. (2.3) as $\overline{Q}, \overline{P}$ are compact in ${\mathbb {R}}^d {\setminus } \{0\}$. The left inequality of (2.5) follows analogously by using that

$$\begin{aligned} |\det B|^j \rho _B(B^{-j} A^i x_0) \le C \rho _A(A^i x_0) = C |\det A|^i \rho _A(x_0), \end{aligned}$$

which completes the proof. $\square$

We also need the following estimates involving parameters $\alpha , \beta \in {\mathbb {R}}$.

#### Lemma 2.5

Let $A, B \in \textrm{GL}(d, {\mathbb {R}})$ be expansive, let $\alpha , \beta \in {\mathbb {R}}{\setminus } \{0\}$, and let $Q, P \subseteq {\mathbb {R}}^d$ be open such that $\overline{Q}, \overline{P}$ are compact in ${\mathbb {R}}^d {\setminus } \{0\}$. If there exists $C > 0$ such that

2.6 $$\begin{aligned} \frac{1}{C} |\det B|^{\beta j} \le |\det A|^{\alpha i} \le C |\det B|^{\beta j} \qquad \text {whenever } A^i Q \cap B^j P \ne \varnothing , \end{aligned}$$

then there exists $N \in {\mathbb {N}}$ such that, for all $i, j \in {\mathbb {Z}}$,

$$\begin{aligned} J_i \subseteq \Big \{ j \in {\mathbb {Z}}:\Big |j - \Big \lfloor \frac{\alpha }{\beta } \, c \, i \Big \rfloor \Big | \le N \Big \} \qquad \text {and} \qquad I_j \subseteq \Big \{ i \in {\mathbb {Z}}:\Big |i - \Big \lfloor \frac{\beta }{\alpha } \, \frac{1}{c} \, j \Big \rfloor \Big | \le N \Big \}, \end{aligned}$$

where $$c = c(A, B):= \ln |\det A| / \ln |\det B|$$.

#### Proof

Taking the logarithm of Eq. (2.6) yields

$$\begin{aligned} \beta j \, \ln (|\det B|) - \ln (C) \le \alpha i \, \ln (|\det A|) \le \beta j \, \ln (|\det B|) + \ln (C), \end{aligned}$$

and thus $\big | \alpha i \ln (|\det A|) - \beta j \ln (|\det B|) \big | \le \ln (C)$. This easily implies that

$$\begin{aligned} \Big | i - j \frac{\beta }{\alpha } \frac{\ln (|\det B|)}{\ln (|\det A|)} \Big | \le \frac{\ln (C)}{|\alpha | \, \ln (|\det A|)}. \end{aligned}$$

Setting $N_1:= \big \lceil \frac{\ln (C)}{|\alpha | \, \ln (|\det A|)} \big \rceil + 1$, it follows that

$$\begin{aligned} I_j \subseteq \Big \{ i \in {\mathbb {Z}}:\Big |i - \Big \lfloor \frac{\beta }{\alpha } \, \frac{1}{c} \, j \Big \rfloor \Big | \le N_1 \Big \} . \end{aligned}$$

The desired inclusion for $J_i$ is obtained analogously with $N_2:= \big \lceil \frac{\ln (C)}{|\beta | \, \ln (|\det B|)} \big \rceil + 1$, which completes the proof by setting $N:= \max \{N_1, N_2\}$. $\square$

#### Corollary 2.6

Let $A, B \in \textrm{GL}(d, {\mathbb {R}})$ be equivalent expansive matrices and $Q, P \subseteq {\mathbb {R}}^d$ open such that $\overline{Q}, \overline{P}$ are compact in ${\mathbb {R}}^d {\setminus } \{0\}$. Then there exists $N \in {\mathbb {N}}$ such that, for all $i, j \in {\mathbb {Z}}$,

$$\begin{aligned} J_i \subseteq \{ j \in {\mathbb {Z}}:|j - \lfloor c \, i \rfloor | \le N \} \qquad \text {and} \qquad I_j \subseteq \{ i \in {\mathbb {Z}}:|i - \lfloor j/c \rfloor | \le N \}, \end{aligned}$$

where $$c = c(A, B):= \ln |\det A| / \ln |\det B|$$.

#### Proof

This follows from Lemmas 2.4 and 2.5 with $\alpha = \beta = 1$. $\square$

Lastly, for a single homogeneous cover $(A^i Q)_{i \in {\mathbb {Z}}}$, we also define the index set

$$\begin{aligned} N_i (A):= \big \{ k \in {\mathbb {Z}}:A^i Q \cap A^k Q \ne \varnothing \big \}. \end{aligned}$$

Note that $N_i (A)$ coincides with the index sets in (2.4) for the choice $A=B$ and $Q = P$. Therefore, the following is a direct consequence of Corollary 2.6.

#### Corollary 2.7

Let $A \in \textrm{GL}(d, {\mathbb {R}})$ be expansive and $Q \subseteq {\mathbb {R}}^d$ open such that $\overline{Q}$ is compact in ${\mathbb {R}}^d {\setminus } \{0\}$. Then there exists $N \in {\mathbb {N}}$ such that, for all $i \in {\mathbb {Z}}$,

$$\begin{aligned} N_i (A) \subseteq \{ j \in {\mathbb {Z}}:|j - i| \le N \}. \end{aligned}$$

## Anisotropic Triebel–Lizorkin spaces

Throughout this section, let $A \in \textrm{GL}(d, {\mathbb {R}})$ be expansive and $\Omega _A$ be an associated ellipsoid.

### Analyzing vectors

A vector $\varphi \in {\mathcal {S}}({\mathbb {R}}^d)$ is called an *A*-*analyzing vector* if its Fourier transform $\widehat{\varphi }$ has compact support

3.1 $$\begin{aligned} \textrm{supp}\,\widehat{\varphi } := \overline{ \{ \xi \in {\mathbb {R}}^d : \widehat{\varphi } (\xi ) \ne 0 \} } \subseteq {\mathbb {R}}^d {\setminus } \{0\} \end{aligned}$$

and satisfies

3.2 $$\begin{aligned} \sup _{i \in {\mathbb {Z}}} | \widehat{\varphi }((A^*)^i \xi ) | > 0, \quad \xi \in {\mathbb {R}}^d {\setminus } \{0\}. \end{aligned}$$

In addition to conditions (3.1) and (3.2), an *A*-analyzing vector $\varphi$ can be chosen to satisfy

3.3 $$\begin{aligned} \sum _{i \in {\mathbb {Z}}} \widehat{\varphi } ((A^*)^i \xi ) = 1 \quad \text {for all} \quad \xi \in {\mathbb {R}}^d {\setminus } \{0\}, \end{aligned}$$

see, e.g., [6, Lemma 3.6] or [12, Remark 2.3]. In most situations, we will choose an *A*-analyzing vector that satisfies (3.3).

### Triebel–Lizorkin spaces

Let $\varphi \in {\mathcal {S}}({\mathbb {R}}^d)$ be a fixed *A*-analyzing vector. For $i \in {\mathbb {Z}}$, let $\varphi _i:= |\det A|^i \varphi (A^i \cdot )$. The (homogeneous) *anisotropic Triebel–Lizorkin space* $\dot{\textbf{F}}^{\alpha }_{p,q}(A)$, with $p \in (0, \infty )$, $q \in (0,\infty ]$ and $\alpha \in {\mathbb {R}}$, is defined as the collection of all $f \in {\mathcal {S}}' / {\mathcal {P}}$ satisfying

3.4 $$\begin{aligned} \Vert f \Vert _{\dot{\textbf{F}}^{\alpha }_{p,q}(A; \varphi )} := \bigg \Vert \bigg ( \sum _{i \in {\mathbb {Z}}} (|\det A|^{\alpha i} \, |f *\varphi _i |)^q \bigg )^{1/q} \bigg \Vert _{L^p} < \infty , \end{aligned}$$

with the usual modifications for $q = \infty$. The space $\dot{\textbf{F}}^{\alpha }_{\infty ,q}(A)$ consists of all $f \in {\mathcal {S}}' / {\mathcal {P}}$ such that

$$\begin{aligned} \Vert f \Vert _{\dot{\textbf{F}}^{\alpha }_{\infty ,q}(A; \varphi )}:= \sup _{\ell \in {\mathbb {Z}}, w \in {\mathbb {R}}^d} \bigg ( \frac{1}{|\det A|^{\ell }} \int _{A^{\ell }\Omega _A + w} \sum _{i = -\ell }^{\infty } (|\det A|^{\alpha i} \, |(f *\varphi _i)(x)|)^q \; d x \bigg )^{1/q} < \infty \end{aligned}$$

if $$q \in (0,\infty )$$, and

$$\begin{aligned} \Vert f \Vert _{\dot{\textbf{F}}^{\alpha }_{\infty ,\infty }(A; \varphi )}:= \sup _{\ell \in {\mathbb {Z}}, w \in {\mathbb {R}}^d} \sup _{i \in {\mathbb {Z}}, i \ge - \ell } \frac{1}{|\det A|^{\ell }} \int _{A^{\ell }\Omega _A + w} |\det A|^{\alpha i} \, |(f *\varphi _i)(x)| \; dx < \infty . \end{aligned}$$

In [6] and the introduction of this paper, the spaces $\dot{\textbf{F}}^{\alpha }_{\infty ,q}(A)$, $q \in (0, \infty )$, are alternatively defined using the cube $[0,1]^d$ instead of an expansive ellipsoid $\Omega _A$. However, it is easily seen that both conditions define the same space, see, e.g., [19, Lemma 2.2]. See also Theorem 3.1 below for the equivalent norm on $\dot{\textbf{F}}^{\alpha }_{\infty ,\infty }(A)$ used in the introduction.

Each space $\dot{\textbf{F}}^{\alpha }_{p,q}(A)$ is continuously embedded into ${\mathcal {S}}' / {\mathcal {P}}$ and is complete with respect to the quasi-norm $\Vert \cdot \Vert _{\dot{\textbf{F}}^{\alpha }_{p,q}}$. In addition, $\dot{\textbf{F}}^{\alpha }_{p,q}(A)$ is independent of the choice of *A*-analyzing vector $\varphi$, with equivalent quasi-norms for different choices. See [6, Section 3] and [4, Section 3.3] for details. We will often simply write $\Vert \cdot \Vert _{\dot{\textbf{F}}^{\alpha }_{p,q}(A)}$ for $\Vert \cdot \Vert _{\dot{\textbf{F}}^{\alpha }_{p,q}(A; \varphi )}$ whenever the precise choice of analyzing vector $\varphi$ does not play a role in our arguments.

For $p, q < \infty$, the space ${\mathcal {S}}_0({\mathbb {R}}^d)$ is a dense subspace of $\dot{\textbf{F}}^{\alpha }_{p,q}(A)$. This fact follows easily from the various atomic and molecular decompositions of $\dot{\textbf{F}}^{\alpha }_{p,q}(A)$, see, e.g., [4, 6, 18, 19].

### Maximal characterizations

For $$\varphi \in {\mathcal {S}} ({\mathbb {R}}^d)$$ and $$i \in {\mathbb {Z}}$$ and $$\eta > 0$$, the associated Peetre-type maximal function $$\varphi ^{**}_{i, \eta } f: {\mathbb {R}}^d \rightarrow [0,\infty ]$$ of $$f \in {\mathcal {S}}' ({\mathbb {R}}^d)$$ is defined by

$$\begin{aligned} \varphi ^{**}_{i, \eta } f (x):= \sup _{z \in {\mathbb {R}}^d} \frac{|(f *\varphi _i) (x+z)|}{(1+\rho _A (A^i z))^{\eta }}, \quad x \in {\mathbb {R}}^d. \end{aligned}$$

The following theorem provides characterizations of Triebel–Lizorkin spaces in terms of Peetre-type maximal functions and will play a key role in Sect. 4. See [18, 19] for proofs.

#### Theorem 3.1

([18, 19]) Let $A \in \textrm{GL}(d, {\mathbb {R}})$ be expansive and $\alpha \in {\mathbb {R}}$. Suppose $\varphi \in {\mathcal {S}}({\mathbb {R}}^d)$ satisfies support conditions (3.1) and (3.2). Then the following norm equivalences hold:

(i) For $$p \in (0,\infty )$$, $$q \in (0, \infty ]$$ and $$\eta > \max \{1/p, 1/q\}$$,
  $$\begin{aligned} \Vert f \Vert _{\dot{\textbf{F}}^{\alpha }_{p,q}(A)} \asymp \bigg \Vert \bigg ( \sum _{i \in {\mathbb {Z}}} (|\det A|^{\alpha i} \varphi ^{**}_{i, \eta } f )^q \bigg )^{1/q} \bigg \Vert _{L^p}, \quad f \in {\mathcal {S}}' /{\mathcal {P}}, \end{aligned}$$
  with the usual modification for $q = \infty$.
(ii) For $q \in (0,\infty )$ and $\eta > 1/q$,
  $$\begin{aligned} \Vert f \Vert _{\dot{\textbf{F}}^{\alpha }_{\infty ,q}(A)}&\asymp \sup _{\ell \in {\mathbb {Z}}, w \in {\mathbb {R}}^d} \bigg ( \frac{1}{|\det A|^{\ell }} \int _{A^{\ell } \Omega _A + w} \sum _{i = - \ell }^{\infty } (|\det A|^{\alpha i} \varphi _{i,\eta }^{**} f (x) )^q \; dx \bigg )^{1/q},\\&\textrm{for} \quad f \in \mathcal {S}' / \mathcal {P}, \end{aligned}$$
(iii) and lastly,
  3.5 $$\begin{aligned} \Vert f \Vert _{\dot{\textbf{F}}^{\alpha }_{\infty ,\infty }(A)} \asymp \sup _{i \in {\mathbb {Z}}} |\det A|^{\alpha i} \Vert f *\varphi _i \Vert _{L^{\infty }}, \quad f \in {\mathcal {S}}' /{\mathcal {P}}. \end{aligned}$$

#### Proof

Assertion (i) is part of [18, Theorem 3.5] and holds for general expansive matrices (cf. [18, Remark 3.6]). Similarly, assertions (ii) and (iii) are part of [19, Theorem 3.3] (cf. [19, Remark 3.4]) and [19, Theorem 4.1], respectively. $\square$

Part (iii) of Theorem 3.1 shows that $\dot{\textbf{F}}^{\alpha }_{\infty , \infty }(A)$ coincides with the anisotropic Besov space $\dot{\textbf{B}}^{\alpha }_{\infty , \infty }(A)$ considered in [3]. In [3, Definition 3.1], the space $\dot{\textbf{B}}^{\alpha }_{\infty , \infty }(A)$ is defined via the right-hand side of the equivalence (3.5).

## Sufficient conditions

This section is devoted to the sufficient conditions of Theorem 1.1 and consists of the proof of the following theorem. A key ingredient used in the proof is the maximal characterization of Triebel–Lizorkin spaces (see Theorem 3.1).

### Theorem 4.1

Let $A, B \in \textrm{GL}(d,{\mathbb {R}})$ be two expansive matrices. If *A* and *B* are equivalent, then $\dot{\textbf{F}}^{\alpha }_{p,q}(A) = \dot{\textbf{F}}^{\alpha }_{p,q}(B)$ for all $p,q \in (0,\infty ]$ and $\alpha \in {\mathbb {R}}$.

### Proof

Let $A, B \in \textrm{GL}(d,{\mathbb {R}})$ be two equivalent expansive matrices. Suppose $\varphi , \psi \in {\mathcal {S}}({\mathbb {R}}^d)$ are analyzing vectors for *A* respectively *B* satisfying additionally Eq. (3.3), i.e., so that $Q:= \bigl \{ \xi \in {\mathbb {R}}^d :\widehat{\varphi } (\xi ) \ne 0 \bigr \}$ and $P:= \bigl \{ \xi \in {\mathbb {R}}^d :\widehat{\psi }(\xi ) \ne 0 \bigr \}$ are relatively compact in ${\mathbb {R}}^d {\setminus } \{0\}$, and

$$\begin{aligned} \sum _{i \in {\mathbb {Z}}} \widehat{\varphi } \bigl ( (A^*)^{-i} \xi \bigr ) = 1 = \sum _{j \in {\mathbb {Z}}} \widehat{\psi } \bigl ( (B^*)^{-j} \xi \bigr ) \quad \text {for all} \quad \xi \in {\mathbb {R}}^d {\setminus } \{0\}. \end{aligned}$$

Then $\bigl ( (A^*)^i Q \bigr )_{i \in {\mathbb {Z}}}$ and $\bigl ( (B^*)^j P \bigr )_{j \in {\mathbb {Z}}}$ are covers of ${\mathbb {R}}^d {\setminus } \{0\}$. Furthermore, a straightforward calculation yields $\widehat{\varphi _i} = \widehat{\varphi } ((A^*)^{-i} \cdot )$ and $\widehat{\psi _j} = \widehat{\psi } ((B^*)^{-j} \cdot )$, and hence $\widehat{\varphi _i} \equiv 0$ outside of $(A^*)^i Q$ and $\widehat{\psi _j} \equiv 0$ outside of $(B^*)^j P$. Since *A* and *B* are equivalent, so are $A^*$ and $B^*$ (cf. Corollary 2.2.)

For fixed $i \in {\mathbb {Z}}$, define $\Psi _i \in {\mathcal {S}}({\mathbb {R}}^d)$ as

$$\begin{aligned} \Psi _i := \sum _{j \in J_i} \psi _j, \end{aligned}$$

where $J_i:= \{ j \in {\mathbb {Z}}: (A^*)^i Q \cap (B^*)^j P \ne \varnothing \}$ is finite by Lemma 2.3. Clearly, $\widehat{\Psi _i} = \sum _{j \in J_i} \widehat{\psi _j} \equiv 1$ on $(A^*)^i Q \supseteq \bigl \{ \xi \in {\mathbb {R}}^d :\widehat{\varphi _i} (\xi ) \ne 0 \bigr \}$ by construction. Therefore,

4.1 $$\begin{aligned} \varphi _i *\Psi _i = \varphi _i \quad \text {for all} \quad i \in {\mathbb {Z}}. \end{aligned}$$

We will use (4.1) to obtain a pointwise estimate of the convolution products $$f *\varphi _i$$, $$i \in {\mathbb {Z}}$$, in terms of the Peetre-type maximal function $$\psi _{j,\eta }^{**}f$$ for a fixed $$\eta > \max \{ 1/p, 1/q \}$$, defined by

$$\begin{aligned} \psi _{j,\eta }^{**}f(x) = \sup _{z \in {\mathbb {R}}^d} \frac{|(f *\psi _j)(x+z)|}{(1+ \rho _B(B^jz))^\eta } \quad \text {for all} \quad x \in {\mathbb {R}}^d; \end{aligned}$$

see Sect. 3.3. For fixed $x \in {\mathbb {R}}^d$, a direct calculation gives

4.2 $$\begin{aligned} |(f *\varphi _i)(x)|&\le \sum _{j \in J_i} |(f *\psi _j*\varphi _i)(x)| \nonumber \\&\le \sum _{j \in J_i} \int _{{\mathbb {R}}^d}\frac{|(f *\psi _j) (x+y)|}{(1+ \rho _B(B^jy))^\eta } \cdot (1+ \rho _B(B^jy))^\eta \, |\varphi _i(-y)| \, dy \nonumber \\&\le \sum _{j \in J_i} \psi _{j,\eta }^{**}f(x) \int _{{\mathbb {R}}^d} (1+ \rho _B(B^jy))^\eta \, |\varphi _i(-y)| \, dy \nonumber \\&= \sum _{j \in J_i} \psi _{j,\eta }^{**}f(x) \int _{{\mathbb {R}}^d} (1+ \rho _B(B^jy))^\eta \, | \det A|^i \, |\varphi (-A^iy)| \, dy \nonumber \\&= \sum _{j \in J_i} \psi _{j,\eta }^{**}f(x) \int _{{\mathbb {R}}^d} (1+ \rho _B(B^jA^{-i}z))^\eta \, |\varphi (-z)| \, dz. \end{aligned}$$

To bound the integral in (4.2), we note that, since $\rho _A,\rho _B$ are equivalent, we have

$$\begin{aligned} \rho _B(B^j A^{-i} z) = |\det B|^j \rho _B(A^{-i}z) \le C |\det B|^j \rho _A(A^{-i}z) = C |\det B|^j |\det A|^{-i} \rho _A(z). \end{aligned}$$

Lemma 2.4 implies that $|\det A|^i \asymp |\det B|^j$ for $j \in J_i$ with implicit constants independent of $i \in {\mathbb {Z}}, j \in J_i$. Consequently, $(1+ \rho _B(B^jA^{-i}z))^\eta \lesssim (1+ \rho _A(z))^\eta$ for all $z \in {\mathbb {R}}^d$. Since $\varphi \in {\mathcal {S}}({\mathbb {R}}^d)$, it follows that for every $N \in {\mathbb {N}}$, there exists $C_N > 0$ such that $|\varphi (z)| \le C_N (1+\rho _A (z))^{-N}$, see, e.g., [2, Section 3]. Combining these observations with (2.3) easily yields

$$\begin{aligned} \int _{{\mathbb {R}}^d} (1+ \rho _B(B^jA^{-i}z))^\eta \, |\varphi (-z)| \, dz \lesssim 1 \end{aligned}$$

with implicit constant independent of $i \in {\mathbb {Z}}$ and $j \in J_i$. Using $|\det A|^i \asymp |\det B|^j$ for $j \in J_i$ once again, it follows thus that

4.3 $$\begin{aligned} |\det A|^{\alpha i} |(f *\varphi _i)(x)| \lesssim \sum _{j \in J_i} |\det B|^{\alpha j} \psi _{j,\eta }^{**}f(x) \quad \text {for all} \quad x \in {\mathbb {R}}^d, \end{aligned}$$

for all $$i \in {\mathbb {Z}}$$.

The remainder of the proof is split into three cases dealing with $p < \infty$, $p = \infty$ and $q < \infty$, and $p = q = \infty$ separately.

**Case 1** $p \in (0, \infty )$. We only prove this case for $q \in (0, \infty )$, since analogous arguments using suprema yield the case for $q = \infty$. Hence, for $q < \infty$, raising (4.3) to the *q*-th power and summing over $i \in {\mathbb {Z}}$ results in

$$\begin{aligned} \sum _{i \in {\mathbb {Z}}} ( |\det A|^{\alpha i} |(f *\varphi _i)(x)|)^q&\lesssim \sum _{i \in {\mathbb {Z}}} \Big (\sum _{j \in J_i} |\det B|^{\alpha j} \psi _{j,\eta }^{**}f(x)\Big )^q \\&\lesssim \sum _{i \in {\mathbb {Z}}} \sum _{j \in J_i} (|\det B|^{\alpha j} \psi _{j,\eta }^{**}f(x))^q, \end{aligned}$$

where we used in the last step that $\sup _{i \in {\mathbb {Z}}}|J_i| < \infty$ by Lemma 2.3. Since Lemma 2.3 also implies $\sup _{j \in {\mathbb {Z}}}|I_j| < \infty$ for $I_j:= \{ i \in {\mathbb {Z}}: (A^*)^iQ \cap (B^*)^jP \ne \varnothing \}$, it follows that

$$\begin{aligned} \sum _{i \in {\mathbb {Z}}} \sum _{j \in J_i} (|\det B|^{\alpha j} \psi _{j,\eta }^{**}f(x))^q&= \sum _{j \in {\mathbb {Z}}} \sum _{i \in I_j} (|\det B|^{\alpha j} \psi _{j,\eta }^{**}f(x))^q \\&\lesssim \sum _{j \in {\mathbb {Z}}} (|\det B|^{\alpha j} \psi _{j,\eta }^{**}f(x))^q. \end{aligned}$$

Consequently, we have

$$\begin{aligned} \Vert f\Vert _{\dot{\textbf{F}}^{\alpha }_{p,q}(A)}= & {} \bigg \Vert \Big ( \sum _{i \in {\mathbb {Z}}} ( |\det A|^{\alpha i} \, |f *\varphi _i|)^q \Big )^{1/q} \bigg \Vert _{L^p} \\\lesssim & {} \bigg \Vert \Big ( \sum _{j \in {\mathbb {Z}}} (|\det B|^{\alpha j} \, \psi _{j,\eta }^{**} f)^q \Big )^{1/q} \bigg \Vert _{L^p} \asymp \Vert f\Vert _{\dot{\textbf{F}}^{\alpha }_{p,q}(B)}, \end{aligned}$$

where the last equivalence follows from Theorem 3.1. Exchanging the roles of *A* and *B* yields the converse inequality and therefore $\dot{\textbf{F}}^{\alpha }_{p,q}(A) = \dot{\textbf{F}}^{\alpha }_{p,q}(B)$ in this case.

**Case 2** $p = \infty$, $q \in (0, \infty )$. Let $\ell \in {\mathbb {Z}}$ be arbitrary. Again, we raise (4.3) to the *q*-th power, sum over $i \ge -\ell$, and use the fact that $\sup _{i \in {\mathbb {Z}}}|J_i| < \infty$. This gives

$$\begin{aligned} \sum _{i = - \ell }^{\infty } ( |\det A|^{\alpha i} |(f *\varphi _i)(x)|)^q&\lesssim \sum _{i = - \ell }^{\infty } \bigg (\sum _{j \in J_i} |\det B|^{\alpha j} \, \psi _{j,\eta }^{**}f(x) \bigg )^q \\&\lesssim \sum _{i = - \ell }^{\infty } \,\, \sum _{j \in J_i} (|\det B|^{\alpha j} \, \psi _{j,\eta }^{**}f(x))^q. \end{aligned}$$

Corollary 2.6 yields the existence of $N_1 \in {\mathbb {N}}$ such that $J_i \subseteq \{j \in {\mathbb {Z}}: |j - \lfloor c i \rfloor | \le N_1 \}$ for all $i \in {\mathbb {Z}}$, where $c = c(A,B):= \ln |\det A| / \ln |\det B|$. Hence, $j \ge \lfloor - c \ell \rfloor - N_1$ for all $j \in \bigcup _{i = - \ell }^\infty J_i$. By setting $\ell _1:= \lfloor c \ell \rfloor + N_1 + 1 \ge -(\lfloor - c \ell \rfloor - N_1)$, we thus obtain

$$\begin{aligned} \sum _{i = - \ell }^{\infty } \,\, \sum _{j \in J_i} (|\det B|^{\alpha j} \, \psi _{j,\eta }^{**}f(x))^q&\le \sum _{j = \lfloor - c \ell \rfloor -N_1}^{\infty } \,\, \sum _{i \in I_j} (|\det B|^{\alpha j} \, \psi _{j,\eta }^{**}f(x))^q \\&\lesssim \sum _{j = - \ell _1}^{\infty } (|\det B|^{\alpha j} \psi _{j,\eta }^{**}f(x))^q, \end{aligned}$$

where in the last step we used that $\sup _{j \in {\mathbb {Z}}}|I_j| < \infty$ for $I_j:= \{ i \in {\mathbb {Z}}: (A^*)^iQ \cap (B^*)^jP \ne \varnothing \}$. In combination, the above two estimates show that, for any $\ell \in {\mathbb {Z}}$,

4.4 $$\begin{aligned} \sum _{i = - \ell }^{\infty } ( |\det A|^{\alpha i} |(f *\varphi _i) (x)|)^q \lesssim \sum _{j = - \ell _1}^{\infty } (|\det B|^{\alpha j} \psi _{j,\eta }^{**}f(x))^q \quad \text {for all} \quad x \in {\mathbb {R}}^d. \end{aligned}$$

Let $\Omega _A, \Omega _B \subseteq {\mathbb {R}}^d$ be the fixed ellipsoids used in the definition of $\rho _A$ resp. $\rho _B$ (cf. Sect. 2.1). Then $A^\ell \Omega _A = \{ x \in {\mathbb {R}}^d: \rho _A(x) < |\det A|^\ell \}$, and thus any $x \in A^\ell \Omega _A$ satisfies

$$\begin{aligned} \rho _B(x) \le C \rho _A(x) < C |\det A|^\ell = C |\det B|^{c \ell } \le |\det B|^{\lfloor c \ell \rfloor +N_2} \end{aligned}$$

with $N_2:= \max \big \{ 1, \, \lceil \ln C / \ln |\det B| \rceil \big \} + N_1 \ge \lceil \ln C / \ln |\det B| \rceil + 1.$ Consequently, we have for all $\ell \in {\mathbb {Z}}$ the inclusion

4.5 $$\begin{aligned} A^\ell \Omega _A \subseteq B^{\lfloor c \ell \rfloor + N_2} \Omega _B = B^{\ell _2} \Omega _B, \qquad \text {where} \qquad \ell _2 := \lfloor c \ell \rfloor + N_2 . \end{aligned}$$

Now let $w \in {\mathbb {R}}^d$ also be arbitrary. Then (4.4) and (4.5) yield

$$\begin{aligned}&\frac{1}{|\det A|^\ell }\int _{A^\ell \Omega _A +w} \sum _{i = - \ell }^{\infty } ( |\det A|^{\alpha i} | (f *\varphi _i)(x)|)^q \; dx \\&\quad \lesssim \frac{1}{|\det A|^\ell } \int _{B^{\ell _2} \Omega _B + w} \sum _{j = - \ell _1}^{\infty } (|\det B|^{\alpha j} \psi _{j,\eta }^{**}f(x))^q \; dx. \end{aligned}$$

Note that $N_1 + 1 \le N_2$ and hence $\ell _1 \le \ell _2$. Therefore, we obtain

4.6 $$\begin{aligned}&\frac{1}{|\det A|^\ell } \int _{A^\ell \Omega _A +w} \sum _{i = - \ell }^{\infty } ( |\det A|^{\alpha i} \, | (f *\varphi _i)(x)|)^q \; dx \nonumber \\&\quad \lesssim \frac{1}{|\det A|^\ell } \int _{B^{\ell _2} \Omega _B + w} \sum _{j = - \ell _2}^{\infty } (|\det B|^{\alpha j} \, \psi _{j,\eta }^{**}f(x))^q \; dx \nonumber \\&\quad \lesssim \frac{1}{|\det B|^{\ell _2}} \int _{B^{\ell _2} \Omega _B + w} \sum _{j = - \ell _2}^{\infty } (|\det B|^{\alpha j} \, \psi _{j,\eta }^{**}f(x))^q \; d x, \end{aligned}$$

where we used in the last step that $|\det A|^{\ell } = |\det B|^{c\ell } \ge |\det B|^{\lfloor c \ell \rfloor } \gtrsim |\det B|^{\ell _2}.$ Taking the *q*-th root and the supremum over $\ell _2, \ell \in {\mathbb {Z}}$ and $w \in {\mathbb {R}}^d$ yields

$$\begin{aligned} \Vert f\Vert _{\dot{\textbf{F}}^{\alpha }_{\infty ,q}(A)}\lesssim & {} \sup _{\ell _2 \in {\mathbb {Z}}, w \in {\mathbb {R}}^d} \bigg ( \frac{1}{|\det B|^{\ell _2}} \int _{B^{\ell _2} \Omega _B + w} \sum _{j = - \ell _2}^{\infty } (|\det B|^{\alpha j} \, \psi _{j,\eta }^{**}f(x))^q \; d x \bigg )^{1/q} \\&\asymp&\Vert f\Vert _{\dot{\textbf{F}}^{\alpha }_{\infty ,q}(B)}, \end{aligned}$$

where the last equivalence follows again from the maximal characterizations of Theorem 3.1. Exchanging the roles of *A* and *B* yield the converse norm estimate, and therefore it yields that $\dot{\textbf{F}}^{\alpha }_{\infty ,q}(A) = \dot{\textbf{F}}^{\alpha }_{\infty ,q}(B)$.

**Case 3** $p = q= \infty$. By Eq. (4.3), it follows that

$$\begin{aligned} \Vert | \det A|^{\alpha i} (f *\varphi _i) \Vert _{L^{\infty }} \lesssim \sum _{j \in J_i} \Vert |\det B|^{\alpha j} \psi ^{**}_{j, \eta } f \Vert _{L^{\infty }} \le \sum _{j \in J_i} \Vert |\det B|^{\alpha j} (f *\psi _j) \Vert _{L^{\infty }} \end{aligned}$$

for $i \in {\mathbb {Z}}$. Combining this with Eq. (3.5) yields

$$\begin{aligned} \Vert f \Vert _{\dot{\textbf{F}}^{\alpha }_{\infty ,\infty }(A; \varphi )}&\asymp \sup _{i \in {\mathbb {Z}}} |\det A|^{\alpha i} \Vert f *\varphi _i \Vert _{L^{\infty }} \\&\lesssim \sup _{i \in {\mathbb {Z}}} \sum _{j \in J_i} |\det B|^{\alpha j} \Vert f *\psi _j \Vert _{L^{\infty }} \\&\lesssim \sup _{i \in {\mathbb {Z}}} \sup _{j \in J_i} |\det B|^{\alpha j} \Vert f *\psi _j \Vert _{L^{\infty }} \\&\le \sup _{j \in {\mathbb {Z}}} |\det B|^{\alpha j} \Vert f *\psi _j \Vert _{L^{\infty }} \\&\asymp \Vert f \Vert _{\dot{\textbf{F}}^{\alpha }_{\infty ,\infty }(B; \psi )}, \end{aligned}$$

where it is used that $\sup _{i \in {\mathbb {Z}}} |J_i| + \sup _{j \in {\mathbb {Z}}} |I_j| < \infty$ by Lemma 2.3. Exchanging the role of *A* and *B* yields $\Vert \cdot \Vert _{\dot{\textbf{F}}^{\alpha }_{\infty ,\infty }(A)} \asymp \Vert \cdot \Vert _{\dot{\textbf{F}}^{\alpha }_{\infty ,\infty }(B)}$, and completes the proof. $\square$

## Necessary conditions

This section is devoted to the proof of the following theorem involving necessary conditions for coincidence of two Triebel–Lizorkin spaces. This theorem corresponds to Theorem 1.2 in the introduction.

### Theorem 5.1

Let $A, B \in \textrm{GL}(d,{\mathbb {R}})$ be expansive matrices, $\alpha , \beta \in {\mathbb {R}}$ and $p_1, p_2, q_1, q_2 \in (0,\infty ]$. If $\dot{\textbf{F}}^{\alpha }_{p_1,q_1}(A) = \dot{\textbf{F}}^{\beta }_{p_2,q_2}(B)$, then $(p_1,q_1,\alpha ) = (p_2,q_2,\beta )$.

Moreover, at least one of the following two cases holds:

(i) *A* and *B* are equivalent, or
(ii) $$\alpha = \beta = 0$$, $$p_1 = p_2 \in (1,\infty )$$, and $$q_1 = q_2 = 2$$.

In the proof of Theorem 5.1, we will often actually use the norm equivalence

5.1 $$\begin{aligned} \Vert f \Vert _{\dot{\textbf{F}}^{\alpha }_{p_1,q_1}(A)} \asymp \Vert f \Vert _{\dot{\textbf{F}}^{\beta }_{p_2,q_2}(B)}, \quad \text {for all} \quad f \in \dot{\textbf{F}}^{\alpha }_{p_1,q_1}(A) = \dot{\textbf{F}}^{\beta }_{p_2,q_2}(B) \end{aligned}$$

rather than the coincidence of the spaces $\dot{\textbf{F}}^{\alpha }_{p_1,q_1}(A) = \dot{\textbf{F}}^{\beta }_{p_2,q_2}(B)$. By a standard density argument, the norm equivalence (5.1) is equivalent to the same condition being satisfied for all elements in a dense subspace. Both facts are contained in the following simple lemma, which will often be used without further mentioning.

### Lemma 5.2

Let $A, B \in \textrm{GL}(d,{\mathbb {R}})$ be expansive matrices, $\alpha , \beta \in {\mathbb {R}}$ and $p_1, p_2, q_1, q_2 \in (0,\infty ]$.

If $\dot{\textbf{F}}^{\alpha }_{p_1,q_1}(A) = \dot{\textbf{F}}^{\beta }_{p_2,q_2}(B)$, then there exists a constant $C \ge 1$ such that

5.2 $$\begin{aligned} \frac{1}{C} \Vert f \Vert _{\dot{\textbf{F}}^{\alpha }_{p_1,q_1}(A)} \le \Vert f \Vert _{\dot{\textbf{F}}^{\beta }_{p_2,q_2}(B)} \le C \Vert f \Vert _{\dot{\textbf{F}}^{\alpha }_{p_1,q_1}(A)} \end{aligned}$$

for all $$f \in \dot{\textbf{F}}^{\alpha }_{p_1,q_1}(A) = \dot{\textbf{F}}^{\beta }_{p_2,q_2}(B)$$.

On the other hand, if $p_1, p_2, q_1, q_2 < \infty$ and Eq. (5.2) holds for all $f \in {\mathcal {S}}_0({\mathbb {R}}^d)$, then $\dot{\textbf{F}}^{\alpha }_{p_1,q_1}(A) = \dot{\textbf{F}}^{\beta }_{p_2,q_2}(B)$.

### Proof

If $\dot{\textbf{F}}^{\alpha }_{p_1,q_1}(A) = \dot{\textbf{F}}^{\beta }_{p_2,q_2}(B)$, then the identity map $\iota : \dot{\textbf{F}}^{\alpha }_{p_1,q_1}(A) \rightarrow \dot{\textbf{F}}^{\beta }_{p_2,q_2}(B), f \mapsto f$ is well-defined. Furthermore, since both $\dot{\textbf{F}}^{\alpha }_{p_1,q_1}(A)$ and $\dot{\textbf{F}}^{\beta }_{p_2,q_2}(B)$ continuously embed into ${\mathcal {S}}'/{\mathcal {P}}= {\mathcal {S}}_0'$ (see Sect. 3.2), it is easy to see that $\iota$ has a closed graph. The norm estimates (5.2) follow therefore by the closed graph theorem, see, e.g., [21, Theorem 2.15]. More precisely, since, by [18, Lemma 5.4] and [19, Lemma 5.6], both $\Vert \cdot \Vert _{\dot{\textbf{F}}^{\alpha }_{p_1,q_1}(A)}$ and $\Vert \cdot \Vert _{\dot{\textbf{F}}^{\beta }_{p_2,q_2}(B)}$ are *r*-norms for $r:= \min \{p,q,1\}$, i.e., both quasi-norms satisfy $\Vert f_1 + f_2 \Vert ^r \le \Vert f_1 \Vert ^r + \Vert f_2 \Vert ^r$, it follows that $\dot{\textbf{F}}^{\alpha }_{p_1,q_1}(A)$ and $\dot{\textbf{F}}^{\beta }_{p_2,q_2}(B)$ are *F*-spaces in the sense of [21, Section 1.8]. Therefore, the closed graph theorem ([21, Theorem 2.15]) applies to $\iota$ and shows that it is bounded, so that

$$\begin{aligned} \Vert f \Vert _{\dot{\textbf{F}}^{\beta }_{p_2,q_2}(B)} \lesssim \Vert f \Vert _{\dot{\textbf{F}}^{\alpha }_{p_1,q_1}(A)} \quad \text {for all} \quad f \in \dot{\textbf{F}}^{\alpha }_{p_1,q_1}(A) = \dot{\textbf{F}}^{\beta }_{p_2,q_2}(B). \end{aligned}$$

The converse estimate is shown in the same way.

For the second part of the lemma, recall that ${\mathcal {S}}_0({\mathbb {R}}^d)$ is norm dense in $\dot{\textbf{F}}^{\alpha }_{p_1,q_1}(A)$ for $p_1,q_1 < \infty$ (cf. Sect. 3.2). Hence, for arbitrary $f \in \dot{\textbf{F}}^{\alpha }_{p_1,q_1}(A)$, there exists a sequence $(f_n)_{n = 1}^\infty$ in ${\mathcal {S}}_0({\mathbb {R}}^d)$ converging to *f* in $\dot{\textbf{F}}^{\alpha }_{p_1,q_1}(A)$. Therefore, if (5.2) holds for all $f_n \in {\mathcal {S}}_0({\mathbb {R}}^d)$, then $(f_n)_{n = 1}^{\infty }$ is a Cauchy sequence in $\dot{\textbf{F}}^{\beta }_{p_2,q_2}(B)$ converging to some $g \in \dot{\textbf{F}}^{\beta }_{p_2,q_2}(B)$. Since convergence in $\dot{\textbf{F}}^{\alpha }_{p_1,q_1}(A)$, respectively $\dot{\textbf{F}}^{\beta }_{p_2,q_2}(B)$, implies weak convergence in ${\mathcal {S}}' /{\mathcal {P}}$ (cf. Sect. 3.2), it follows that $f = g \in \dot{\textbf{F}}^{\beta }_{p_2,q_2}(B)$. This shows $\dot{\textbf{F}}^{\alpha }_{p_1,q_1}(A) \subseteq \dot{\textbf{F}}^{\beta }_{p_2,q_2}(B)$. The reverse inclusion is shown similarly. $\square$

### Preparations and notation

This section sets up some essential objects and notation that will be used for the proof of Theorem 5.1. This notation will be kept throughout Sect. 5.

Let $A, B \in \textrm{GL}(d, {\mathbb {R}})$ be expansive matrices. Fix analyzing vectors $\varphi \in {\mathcal {S}}({\mathbb {R}}^d)$ and $\psi \in {\mathcal {S}}({\mathbb {R}}^d)$ satisfying Eq. (3.3) for *A* and *B*, respectively. Then

$$\begin{aligned} Q:= \bigl \{ \xi \in {\mathbb {R}}^d :\widehat{\varphi } (\xi ) \ne 0 \bigr \} \qquad \text {and} \qquad P:= \bigl \{ \xi \in {\mathbb {R}}^d :\widehat{\psi }(\xi ) \ne 0 \bigr \}, \end{aligned}$$

are open, relatively compact sets in ${\mathbb {R}}^d {\setminus } \{0\}$. In the following, we mainly consider the covers $\bigl ( (A^*)^i Q \bigr )_{i \in {\mathbb {Z}}}$ and $\bigl ( (B^*)^j P \bigr )_{j \in {\mathbb {Z}}}$ of ${\mathbb {R}}^d {\setminus } \{ 0 \}$. In particular, we will take the sets $I_j$ and $J_i$ defined in Eq. (2.4) to be defined with respect to these two coverings; explicitly, this means

5.3 $$\begin{aligned} I_j := \big \{ k \in {\mathbb {Z}}:(A^*)^k Q \cap (B^*)^j P \ne \varnothing \big \} \quad \text {and} \quad J_i := \big \{ k \in {\mathbb {Z}}:(B^*)^k P \cap (A^*)^i Q \ne \varnothing \big \} \end{aligned}$$

for $i, j \in {\mathbb {Z}}$. Furthermore, for $i \in {\mathbb {Z}}$, we will use the index sets

$$\begin{aligned}{} & {} N_i (A^*):= \big \{ j \in {\mathbb {Z}}:(A^*)^i Q \cap (A^*)^j Q \ne \varnothing \big \} \quad \text {and} \\{} & {} N_i (B^*):= \big \{ j \in {\mathbb {Z}}:(B^*)^i P \cap (B^*)^j P \ne \varnothing \big \}. \end{aligned}$$

As shown in Corollary 2.7, there exists $N = N(A,B,Q,P) \in {\mathbb {N}}$ satisfying

5.4 $$\begin{aligned} N_i(A^*) \cup N_i(B^*) \subseteq \{ j \in {\mathbb {Z}}:|j - i| \le N \} \qquad \text {for all} \quad i \in {\mathbb {N}}. \end{aligned}$$

Throughout, we fix such an *N* and define the functions

$$\begin{aligned} \Phi := \sum _{i=-N}^{N} \varphi _i \qquad \text {and} \qquad \Psi := \sum _{j=-N}^{N} \psi _j. \end{aligned}$$

In view of Eq. (3.3) and because $(A^*)^i Q \cap Q \ne \varnothing$ can only hold if $|i| \le N$ by Eq. (5.4), it follows that $\widehat{\Phi } \equiv 1$ on *Q* and $\widehat{\Psi } \equiv 1$ on *P*. In particular, $\Phi$ and $\Psi$ satisfy the analyzing vector conditions (3.1) and (3.2) for *A* and *B*, respectively.

In addition to the above, we fix throughout a non-zero function $\phi \in {\mathcal {S}}({\mathbb {R}}^d)$ satisfying $\widehat{\phi } \ge 0$ and $\textrm{supp}\,\widehat{\phi } \subseteq B_1(0)$. For $\delta > 0$, define

5.5 $$\begin{aligned} \phi _\delta (x) := \delta ^d \, \phi (\delta x). \end{aligned}$$

Then $\widehat{\phi _\delta }(\xi ) = \widehat{\phi }(\xi /\delta )$ and thus $\textrm{supp}\,\widehat{\phi _\delta } \subseteq B_\delta (0)$. In order to distinguish an isotropic dilation as in (5.5) from an anisotropic dilation, we use a Greek letter subscript to denote an isotropic dilation.

### Norm estimates for auxiliary functions

This subsection consists of two estimates of the Triebel–Lizorkin norms of functions with specific Fourier support. These functions play an essential role in our proof of Theorem 5.1 and will be used in the following subsections.

#### Proposition 5.3

Let $A \in \textrm{GL}(d,{\mathbb {R}})$ be expansive, $\alpha \in {\mathbb {R}}$ and $p, q \in (0, \infty ]$. If $f \in {\mathcal {S}}({\mathbb {R}}^d)$ satisfies $\textrm{supp}\,\widehat{f} \subseteq (A^*)^{i_0}Q$ for $i_0 \in {\mathbb {Z}}$, then

5.6 $$\begin{aligned} \Vert f\Vert _{\dot{\textbf{F}}^{\alpha }_{p,q}(A)} \asymp |\det A|^{\alpha i_0} \Vert f\Vert _{L^p}, \end{aligned}$$

with an implicit constant independent of $i_0$ and *f*.

#### Proof

With notation as in Sect. 5.1, we start by collecting some basic facts about the convolutions $f *\varphi _i$ and $f *\Phi _{i_0}$ for *f* as in the statement of the proposition. First, note that since $\widehat{\varphi _i} \equiv 0$ outside of $(A^*)^{i}Q$, it follows that $f *\varphi _i = 0$ whenever $(A^*)^{i_0}Q \cap (A^*)^{i}Q = \varnothing$, which holds whenever $|i-i_0| > N$, by Eq. (5.4). Therefore,

5.7 $$\begin{aligned} f *\varphi _i \equiv 0 \qquad \text {for }|i-i_0|> N. \end{aligned}$$

For the convolution $f *\Phi _{i_0}$ observe that $\widehat{\Phi _{i_0}} \equiv 1$ on $(A^*)^{i_0}Q$ by construction, and therefore

5.8 $$\begin{aligned} f *\Phi _{i_0} = {\mathcal {F}}^{-1}(\widehat{f} \cdot \widehat{\Phi _{i_0}}) = f. \end{aligned}$$

In the remainder of this proof, we deal with the cases $p < \infty$, $p = \infty$ and $q < \infty$, and $p = q = \infty$ separately.

**Case 1** $p \in (0, \infty )$. For the upper bound in Eq. (5.6), we use (5.7) to obtain

$$\begin{aligned} \Vert f \Vert _{\dot{\textbf{F}}^{\alpha }_{p,q}(A;\varphi )} = \bigg \Vert \Big \Vert \Big ( |\det A|^{\alpha i} |f *\varphi _i| \Big )_{i \in {\mathbb {Z}}} \Big \Vert _{\ell ^q} \bigg \Vert _{L^p} \lesssim _{p,q,N} \sum _{i = i_0-N}^{i_0 + N} |\det A|^{\alpha i} \Vert f *\varphi _i \Vert _{L^p}. \end{aligned}$$

If $p \in [1,\infty )$, then Young’s inequality shows

$$\begin{aligned} \Vert f *\varphi _i \Vert _{L^p} \le \Vert f \Vert _{L^p} \Vert \varphi _i \Vert _{L^1} \lesssim _{\varphi } \Vert f \Vert _{L^p}. \end{aligned}$$

If $p \in (0,1)$, then, since $\textrm{supp}\,\widehat{f}, \textrm{supp}\,\widehat{\varphi _i} \subseteq \bigcup _{\ell = -N}^N (A^*)^{i_0 + \ell } \overline{Q}$ for $|i - i_0| \le N$, an application of Corollary A.2 yields

$$\begin{aligned} \Vert f *\varphi _i \Vert _{L^p}&\lesssim _{A,Q,N,p} |\det A|^{i_0 \left( \frac{1}{p} - 1\right) } \Vert f \Vert _{L^p} \Vert \varphi _i \Vert _{L^p} \\&= |\det A|^{(i_0 - i) \left( \frac{1}{p} - 1\right) } \Vert f \Vert _{L^p} \Vert \varphi \Vert _{L^p} \\&\lesssim _{A,N,\varphi ,p} \Vert f \Vert _{L^p}. \end{aligned}$$

Consequently, for arbitrary $p \in (0, \infty )$

$$\begin{aligned} \Vert f \Vert _{\dot{\textbf{F}}^{\alpha }_{p,q}(A;\varphi )} \lesssim _{p,q,N} \sum _{i = i_0-N}^{i_0 + N} |\det A|^{\alpha i} \Vert f *\varphi _i \Vert _{L^p} \lesssim _{A,Q,N, \alpha , p,\varphi } |\det A|^{\alpha i_0} \Vert f \Vert _{L^p}, \end{aligned}$$

which proves the desired upper bound.

For the lower bound, using Eq. (5.8) and the equivalence $\Vert \cdot \Vert _{\dot{\textbf{F}}^{\alpha }_{p,q}(A; \varphi )} \asymp \Vert \cdot \Vert _{\dot{\textbf{F}}^{\alpha }_{p,q}(A; \Phi )}$ (see Sect. 3.2) gives

$$\begin{aligned} \Vert f \Vert _{\dot{\textbf{F}}^{\alpha }_{p,q}(A;\varphi )}&\asymp _{\varphi ,N,p,q,A,\alpha } \Vert f \Vert _{\dot{\textbf{F}}^{\alpha }_{p,q}(A;\Phi )} = \bigg \Vert \Big \Vert \Big ( |\det A|^{\alpha i} |f *\Phi _i| \Big )_{i \in {\mathbb {Z}}} \Big \Vert _{\ell ^q} \bigg \Vert _{L^p} \\&\ge |\det A|^{\alpha i_0} \Vert f *\Phi _{i_0} \Vert _{L^p} = |\det A|^{\alpha i_0} \Vert f \Vert _{L^p}, \end{aligned}$$

as required.

**Case 2** $p = \infty$, $q \in (0, \infty )$. As in the previous case, we use Eq. (5.7) for the upper estimate. This yields

$$\begin{aligned} \Vert f\Vert _{\dot{\textbf{F}}^{\alpha }_{\infty ,q}(A;\varphi )}&= \sup _{\ell \in {\mathbb {Z}}, w \in {\mathbb {R}}^d} \bigg ( \frac{1}{|\det A|^{\ell }} \int _{A^{\ell } \Omega _A + w} \sum _{i = - \ell }^{\infty } (|\det A|^{\alpha i} |(f *\varphi _i)(x)|)^q \; dx \bigg )^{1/q} \\&\le \sup _{\ell \in {\mathbb {Z}}, w \in {\mathbb {R}}^d} \bigg ( \frac{1}{|\det A|^{\ell }} \int _{A^{\ell } \Omega _A + w} \sum _{i = i_0 -N}^{i_0+N} (|\det A|^{\alpha i} |(f *\varphi _i)(x)|)^q \; dx \bigg )^{1/q} \\&\lesssim _{A, N, q, \alpha } |\det A|^{\alpha i_0} \sum _{i = i_0 -N}^{i_0+N} \Vert f *\varphi _i\Vert _{L^\infty } \\&\le |\det A|^{\alpha i_0} \sum _{i = i_0 -N}^{i_0+N} \Vert f \Vert _{L^\infty } \Vert \varphi _i\Vert _{L^1} \\&\lesssim _{N, \varphi } |\det A|^{\alpha i_0} \Vert f \Vert _{L^\infty }. \end{aligned}$$

For the lower bound, we use the continuous embedding $\dot{\textbf{F}}^{\alpha }_{\infty ,q}(A) \hookrightarrow \dot{\textbf{F}}^{\alpha }_{\infty ,\infty }(A)$ (cf. [19, Theorem 4.1]), the norm equivalence $\Vert \cdot \Vert _{\dot{\textbf{F}}^{\alpha }_{\infty ,\infty }(A; \varphi )} \asymp \Vert \cdot \Vert _{\dot{\textbf{F}}^{\alpha }_{\infty ,\infty }(A; \Phi )}$, and Eq. (3.5) to obtain

$$\begin{aligned} \Vert f\Vert _{\dot{\textbf{F}}^{\alpha }_{\infty ,q}(A;\varphi )}&\gtrsim _{\varphi ,q,A,\alpha } \Vert f\Vert _{\dot{\textbf{F}}^{\alpha }_{\infty ,\infty }(A;\varphi )} \asymp _{\varphi , N, A,\alpha } \Vert f\Vert _{\dot{\textbf{F}}^{\alpha }_{\infty ,\infty }(A;\Phi )} \\&\asymp _{\varphi ,N,A,\alpha } \sup _{i \in {\mathbb {Z}}} |\det A|^{\alpha i} \Vert f *\Phi _i \Vert _{L^\infty } \\&\ge |\det A|^{\alpha i_0} \Vert f *\Phi _{i_0} \Vert _{L^\infty } = |\det A|^{\alpha i_0} \Vert f \Vert _{L^\infty }, \end{aligned}$$

where the final step follows from Eq. (5.8).

**Case 3** $p =q = \infty$. The lower bound $\Vert f\Vert _{\dot{\textbf{F}}^{\alpha }_{\infty ,\infty }(A;\varphi )} \gtrsim |\det A|^{\alpha i_0} \Vert f \Vert _{L^\infty }$ has been shown in the previous case already. For the reverse, observe that (3.5) and (5.7) yield

$$\begin{aligned} \Vert f\Vert _{\dot{\textbf{F}}^{\alpha }_{\infty ,\infty }(A;\varphi )}&\asymp \sup _{i \in {\mathbb {Z}}} |\det A|^{\alpha i} \Vert f *\varphi _i \Vert _{L^\infty } = \sup _{|i - i_0| \le N} |\det A|^{\alpha i} \Vert f *\varphi _i \Vert _{L^\infty } \\&\le \sup _{|i - i_0| \le N} |\det A|^{\alpha i} \Vert f\Vert _{L^\infty } \Vert \varphi _i \Vert _{L^1} \lesssim _{N, A, \alpha , \varphi } |\det A|^{\alpha i_0} \Vert f\Vert _{L^\infty }, \end{aligned}$$

which completes the proof. $\square$

The following simple consequence is what actually will be used in obtaining necessary conditions for the coincidence of Triebel–Lizorkin spaces.

#### Corollary 5.4

Let $A, B \in \textrm{GL}(d,{\mathbb {R}})$ be expansive, $\alpha , \beta \in {\mathbb {R}}$ and $p_1, p_2, q_1, q_2 \in (0,\infty ]$.

Suppose that $\dot{\textbf{F}}^{\alpha }_{p_1,q_1}(A) = \dot{\textbf{F}}^{\beta }_{p_2,q_2}(B)$. If $(A^*)^i Q \cap (B^*)^j P \ne \varnothing$ for some $i, j \in {\mathbb {Z}}$, then there exists $\delta _0 = \delta _0(i,j) > 0$ such that for all $0 < \delta \le \delta _0$, it holds that

$$\begin{aligned} |\det A|^{\alpha i} \delta ^{d (1-1/p_1)} \asymp |\det B|^{\beta j} \delta ^{d (1-1/p_2)}, \end{aligned}$$

where the implicit constants are independent of $i, j, \delta , \delta _0$.

#### Proof

Since $(A^*)^i Q \cap (B^*)^j P \ne \varnothing$ is open, there exists $\eta \in {\mathbb {R}}^d$ and $\delta _0 > 0$ such that $B_{\delta _0}(\eta ) \subseteq (A^*)^i Q \cap (B^*)^j P$. For a fixed $0 < \delta \le \delta _0$, define $f^{(\delta )}:= M_\eta \phi _\delta$. Then

$$\begin{aligned} \textrm{supp}\,\widehat{f^{(\delta )}} = \textrm{supp}\,T_\eta \widehat{\phi _\delta } \subseteq B_{\delta }(\eta ). \end{aligned}$$

Using the estimates of Proposition 5.3 for $\Vert f^{(\delta )}\Vert _{\dot{\textbf{F}}^{\alpha }_{p_1,q_1}(A)}$ and $\Vert f^{(\delta )}\Vert _{\dot{\textbf{F}}^{\beta }_{p_2,q_2}(B)}$ yields

$$\begin{aligned} |\det A|^{\alpha i} \delta ^{d (1-1/p_1)}&= |\det A|^{\alpha i} \, \Vert f^{(\delta )}\Vert _{L^{p_1}} \asymp \Vert f^{(\delta )}\Vert _{\dot{\textbf{F}}^{\alpha }_{p_1,q_1}(A)} \asymp \Vert f^{(\delta )}\Vert _{\dot{\textbf{F}}^{\beta }_{p_2,q_2}(B)} \\&\asymp |\det B|^{\beta j} \delta ^{d (1-1/p_2)}, \end{aligned}$$

with implicit constants independent of $i,j, \delta , \delta _0$. $\square$

The following proposition provides a more technical version of Proposition 5.3 and involves a linear combination of functions with Fourier supports in $(A^*)^{i_k} Q$ for suitable points $i_k \in {\mathbb {Z}}$. The proof strategy resembles the one of Proposition 5.3, but requires various technical modifications.

#### Proposition 5.5

Let $A \in \textrm{GL}(d,{\mathbb {R}})$ be expansive, $\alpha \in {\mathbb {R}}$ and $p, q \in (0, \infty ]$. For $K \in {\mathbb {N}}$, let $i_1, \dots , i_K \in {\mathbb {Z}}$ be increasing with $|i_k - i_{k'}| > 2N$ if $k \ne k'$, where $N \in {\mathbb {N}}$ is as in Eq. (5.4).

Suppose there exists $\delta _0 > 0$ and points $\eta _1, \dots , \eta _K \in {\mathbb {R}}^d$ such that:

- $$B_{\delta _0}(\eta _k) \subseteq (A^*)^{i_k}Q$$ for all $$k=1, \dots , K$$,
- $$|\phi (x)| \ge \frac{1}{2}|\phi (0)|$$ for all $$x \in \delta _0 A^{-i_1}\Omega _A$$.

Then, for all $0 < \delta \le \delta _0$ and $c \in {\mathbb {C}}^K$, the function $f = \sum _{k=1}^K c_k M_{\eta _k} \phi _\delta$ satisfies

5.9 $$\begin{aligned} \Vert f \Vert _{\dot{\textbf{F}}^{\alpha }_{p,q}(A)} \asymp \delta ^{d (1 -1/p)} \Big \Vert \Big (|\det A|^{\alpha i_k} \, |c_k|\Big )_{k =1}^K \Big \Vert _{\ell ^q}, \end{aligned}$$

where the implicit constant is independent of $K, c, \delta , \delta _0, \eta _1, \dots , \eta _K, i_1, \dots , i_K$.

#### Remark 5.6

Assumption (b) in Proposition 5.5 is only needed for the case $p = \infty$, $q \in (0, \infty )$.

#### Proof

Using the notation from Sect. 5.1, we first state some basic observations for $f *\varphi _i$ and $f *\Phi _{i_k}$ with *f* as in the statement. First, note that by assumption (a) it follows that $\textrm{supp}\,T_{\eta _k}\widehat{\phi _\delta } \subseteq (A^*)^{i_k}Q$ for all $k=1, \dots , K$. Since $\widehat{\varphi _i} \equiv 0$ outside of $(A^*)^{i}Q$, this implies

$$\begin{aligned} M_{\eta _k}[\phi _\delta ] *\varphi _i = {\mathcal {F}}^{-1} (T_{\eta _k}[\widehat{\phi _\delta }] \cdot \widehat{\varphi _i}) = 0 \qquad \text {for } |i - i_k| > N, \end{aligned}$$

as $(A^*)^{i_k}Q \cap (A^*)^{i}Q = \varnothing$ for $|i - i_k| > N$ by Eq. (5.4). Furthermore, note that for fixed $i \in {\mathbb {Z}}$, there can be at most one point $i_k$ such that $|i - i_k| \le N$ due to the pairwise minimal distance between the chosen points $i_1,\ldots , i_K$. This implies that

5.10 $$\begin{aligned} f *\varphi _i = \sum _{k=1}^K c_k \cdot (M_{\eta _k} [\phi _\delta ] *\varphi _i) = {\left\{ \begin{array}{ll} c_k \cdot (M_{\eta _k} [\phi _\delta ] *\varphi _i), &{}\quad \text {if } |i - i_k| \le N,\\ 0, &{}\quad \text {otherwise.} \end{array}\right. } \end{aligned}$$

Second, for $f *\Phi _{i_k}$, observe that $\widehat{\Phi _{i_k}} \equiv 0$ outside of $\bigcup _{i= i_k -N}^{i_k+N}(A^*)^{i}Q$ for all $k = 1, \dots , K$ by construction of $\Phi$. Since $|i_k - i_{k'}| > 2N$ for $k \ne k'$, it follows by Eq. (5.4) that

$$\begin{aligned} (A^*)^{i_{k'}}Q \cap \bigcup _{i= i_k -N}^{i_k+N}(A^*)^{i}Q = \varnothing , \quad \text {for} \quad k \ne k'. \end{aligned}$$

This implies $M_{\eta _{k'}} [\phi _\delta ] *\Phi _{i_k} = {\mathcal {F}}^{-1}(T_{\eta _{k'}} [\widehat{\phi _\delta }] \cdot \widehat{\Phi _{i_k}}) = 0$ for $k \ne k'$. Since also $\widehat{\Phi _{i_k}} \equiv 1$ on $(A^*)^{i_k}Q \supseteq \textrm{supp}\,T_{\eta _k}\widehat{\phi _\delta }$, necessarily

5.11 $$\begin{aligned} f *\Phi _{i_k} = \sum _{k'=1}^K c_{k'} \cdot (M_{\eta _{k'}} [\phi _\delta ] *\Phi _{i_k}) = c_kM_{\eta _k} \phi _\delta \qquad \text {for } k= 1, \dots K. \end{aligned}$$

The remainder of the proof is divided into three cases and deals with $p < \infty$, $p = \infty$ and $q < \infty$, and $p = q = \infty$ separately.

**Case 1** $p \in (0, \infty )$. For the upper bound in Eq. (5.9), set $M = \frac{d}{p} + 1$. Then, in view of Eq. (5.10), an application of Lemma A.3 with $\ell = i_k$ shows that

$$\begin{aligned} |f *\varphi _i (x)|&= |c_k| \cdot |(M_{\eta _k} [\phi _\delta ] *\varphi _i)(x)| \le |c_k| \cdot (|\phi _\delta | *|\varphi _i|) (x) \\&\lesssim _{N,A,d,p,Q,\phi ,\varphi } |c_k| \delta ^d (1 + |\delta x|)^{-M} \end{aligned}$$

whenever $| i - i_k| \le N$. On the other hand, $f *\varphi _i = 0$ if $|i - i_k| > N$ for all $k = 1,\dots ,K$. Therefore, for all $x \in {\mathbb {R}}^d$,

$$\begin{aligned}&\Big \Vert \Big ( |\det A|^{\alpha i} |f *\varphi _i(x)| \Big )_{i \in {\mathbb {Z}}} \Big \Vert _{\ell ^q} \\&\quad \le \bigg ( \sum _{k = 1}^{K} \sum _{i= i_k -N}^{i_k +N} (|\det A|^{\alpha i} \, |f *\varphi _i(x)|)^q \bigg )^{1/q} \\&\quad \lesssim _{N,A,Q,d,p,q,\phi ,\varphi ,\alpha } \bigg ( \sum _{k = 1}^{K} \big ( |\det A|^{\alpha i_k} \, |c_k| \, \delta ^d \, (1 + |\delta x|)^{-M} \big )^q \bigg )^{1/q} \\&\quad = \delta ^d \, (1 + |\delta x|)^{-M} \, \Big \Vert \Big (|\det A|^{\alpha i_k} |c_k|\Big )_{k =1}^K \Big \Vert _{\ell ^q}, \end{aligned}$$

with the usual modification of the argument for $q = \infty$. Consequently, this yields

$$\begin{aligned} \Vert f \Vert _{\dot{\textbf{F}}^{\alpha }_{p,q}(A;\varphi )}&= \bigg \Vert \Big \Vert \Big ( |\det A|^{\alpha i} |f *\varphi _i| \Big )_{i \in {\mathbb {Z}}} \Big \Vert _{\ell ^q} \bigg \Vert _{L^p} \\&\lesssim _{N,A,Q,d,p,q,\phi ,\varphi ,\alpha } \bigg ( \int _{{\mathbb {R}}^d} (\delta ^d \, (1 + |\delta x|)^{-M})^p \, dx \bigg )^{1/p} \Big \Vert \Big ( |\det A|^{\alpha i_k} |c_k| \Big )_{k =1}^K \Big \Vert _{\ell ^q} \\&\lesssim _{d,p} \delta ^{d (1- 1/p)} \, \Big \Vert \Big (|\det A|^{\alpha i_k} |c_k|\Big )_{k =1}^K \Big \Vert _{\ell ^q}, \end{aligned}$$

where the last step used that $M > \frac{d}{p}$, so that $\int _{{\mathbb {R}}^d} (1 + |x|)^{-Mp} \, dx < \infty$.

For the lower bound, we use the equivalence $\Vert \cdot \Vert _{\dot{\textbf{F}}^{\alpha }_{p,q}(A; \varphi )} \asymp \Vert \cdot \Vert _{\dot{\textbf{F}}^{\alpha }_{p,q}(A; \Phi )}$ and Eq. (5.11) to obtain

$$\begin{aligned} \Vert f \Vert _{\dot{\textbf{F}}^{\alpha }_{p,q}(A;\varphi )}&\asymp _{A,p,q,\alpha ,\varphi ,N} \bigg \Vert \Big \Vert \Big ( |\det A|^{\alpha i} \, |f *\Phi _i| \Big )_{i \in {\mathbb {Z}}} \Big \Vert _{\ell ^q} \bigg \Vert _{L^p} \\&\ge \bigg \Vert \Big \Vert \Big ( |\det A|^{\alpha i_k} \, |f *\Phi _{i_k}| \Big )_{k =1}^K \Big \Vert _{\ell ^q} \bigg \Vert _{L^p} \\&= \Vert \phi _\delta \Vert _{L^p} \Big \Vert \Big ( |\det A|^{\alpha i_k} \, |c_k| \Big )_{k =1}^K \Big \Vert _{\ell ^q} \\&\gtrsim _{\phi ,p} \delta ^{d (1- 1/p)} \Big \Vert \Big ( |\det A|^{\alpha i_k} \, |c_k| \Big )_{k =1}^K \Big \Vert _{\ell ^q}, \end{aligned}$$

as required.

**Case 2** $p = \infty$, $q \in (0, \infty )$. The upper estimate in Eq. (5.9) follows by an application of Eq. (5.10):

$$\begin{aligned}&\Vert f\Vert _{\dot{\textbf{F}}^{\alpha }_{\infty ,q}(A;\varphi )} \\&\quad \le \sup _{\ell \in {\mathbb {Z}}, w \in {\mathbb {R}}^d} \bigg ( \frac{1}{|\det A|^{\ell }} \int _{A^{\ell } \Omega _A +w} \sum _{k= 1}^{K} \sum _{i=i_k -N}^{i_k +N} \big ( |\det A|^{\alpha i} \, |c_k| \, (|\phi _\delta | *|\varphi _{i}|) (x) \big )^q \; dx \bigg )^{1/q} \\&\le \bigg ( \sum _{k= 1}^{K} \sum _{i=i_k -N}^{i_k +N} \big ( |\det A|^{\alpha i} \, |c_k| \, \Vert |\phi _\delta | *|\varphi _{i}| \Vert _{L^\infty } \big )^q \bigg )^{1/q} \\&\le \bigg ( \sum _{k= 1}^{K} \sum _{i=i_k -N}^{i_k +N} \big ( |\det A|^{\alpha i} \, |c_k| \, \Vert \phi _\delta \Vert _{L^\infty } \, \Vert \varphi _{i} \Vert _{L^1} \big )^q \bigg )^{1/q} \\&\lesssim _{A, N, q, \alpha } \delta ^d \, \Vert \phi \Vert _{L^\infty } \Vert \varphi \Vert _{L^1} \bigg (\sum _{k= 1}^{K} (|\det A|^{\alpha i_k}|c_k|)^q \bigg )^{1/q}. \end{aligned}$$

For the reverse inequality, we again use the *A*-analyzing vector $\Phi$. We start by taking $w = 0$ and $\ell = - i_1$ in the supremum below. Note that this choice ensures that the sum over $i \ge - \ell = i_1$ includes all $i_k$ for $k=1, \dots , K$ as they are increasing. By Eq. (5.11), it follows that

$$\begin{aligned}&\Vert f\Vert _{\dot{\textbf{F}}^{\alpha }_{\infty ,q}(A;\varphi )} \\&\quad \asymp _{A,q,\alpha ,\varphi ,N} \sup _{\ell \in {\mathbb {Z}}, w \in {\mathbb {R}}^d} \bigg ( \frac{1}{|\det A|^{\ell }} \int _{A^{\ell } \Omega _A + w} \sum _{i = - \ell }^{\infty } (|\det A|^{\alpha i} \, |(f *\Phi _i) (x)|)^q \, dx \bigg )^{1/q} \\&\ge \bigg ( \frac{1}{|\det A|^{-i_1}} \int _{A^{-i_1} \Omega _A} \sum _{k= 1}^{K} (|\det A|^{\alpha i_k} \, |(f *\Phi _{i_k}) (x)|)^q \, dx \bigg )^{1/q} \\&= \bigg ( \frac{1}{|\det A|^{-i_1}} \int _{A^{-i_1} \Omega _A} \sum _{k= 1}^{K} (|\det A|^{\alpha i_k} \, |c_k| \, |\phi _\delta (x)|)^q \, dx \bigg )^{1/q}\\&\ge \min _{x \in \delta A^{-i_1} \Omega _A} \delta ^d \, |\phi (x)| \, \Big \Vert \Big ( |\det A|^{\alpha i_k} \, |c_k| \Big )_{k =1}^K \Big \Vert _{\ell ^q} \\&\ge \delta ^d \, \frac{1}{2} \, |\phi (0)| \Big \Vert \Big ( |\det A|^{\alpha i_k} \, |c_k| \Big )_{k =1}^K \Big \Vert _{\ell ^q}, \end{aligned}$$

where we used the assumption $|\phi (x)| \ge \frac{1}{2} |\phi (0)|$ for $x \in \delta _0 A^{-i_1} \Omega _A$ in the last step. Furthermore, note that $\phi (0) > 0$ since $\widehat{\phi } \ge 0$ and $\widehat{\phi } \not \equiv 0$, so that $\phi (0) = \int _{{\mathbb {R}}^d} \widehat{\phi }(\xi ) \, d \xi > 0$.

**Case 3** $p = q = \infty$. Eqs. (3.5) and (5.10) allow to obtain the upper bound:

$$\begin{aligned} \Vert f\Vert _{\dot{\textbf{F}}^{\alpha }_{\infty ,\infty }(A;\varphi )}&\asymp \sup _{i \in {\mathbb {Z}}} |\det A|^{\alpha i}\Vert f *\varphi _i \Vert _{L^\infty } \\&= \sup _{k=1, \dots , K} \,\, \sup _{|i - i_k| \le N} |\det A|^{\alpha i} \Vert f *\varphi _i \Vert _{L^\infty }\\&\le \sup _{k=1, \dots , K} \,\, \sup _{|i - i_k| \le N} |\det A|^{\alpha i} \, |c_k| \, \Vert \phi _\delta \Vert _{L^\infty } \, \Vert \varphi _{i}\Vert _{L^1} \\&\lesssim _{N, A, \alpha , \phi , \varphi } \delta ^d \Big \Vert \Big ( |\det A|^{\alpha i_k} \, |c_k| \Big )_{k = 1}^K \Big \Vert _{\ell ^{\infty }}. \end{aligned}$$

For the lower bound, combining the norm equivalence $\Vert \cdot \Vert _{\dot{\textbf{F}}^{\alpha }_{\infty ,\infty }(A; \varphi )} \asymp \Vert \cdot \Vert _{\dot{\textbf{F}}^{\alpha }_{\infty ,\infty }(A; \Phi )}$ and Eqs. (3.5 and (5.11) yields

$$\begin{aligned} \Vert f\Vert _{\dot{\textbf{F}}^{\alpha }_{\infty ,\infty }(A;\varphi )}&\asymp _{A,\varphi ,N,\alpha } \Vert f\Vert _{\dot{\textbf{F}}^{\alpha }_{\infty ,\infty }(A;\Phi )} \asymp \sup _{i \in {\mathbb {Z}}} |\det A|^{\alpha i} \, \Vert f *\Phi _i \Vert _{L^\infty } \\&\ge \sup _{k=1, \dots , K} |\det A|^{\alpha i_k} \, \Vert f *\Phi _{i_k} \Vert _{L^\infty } = \sup _{k=1, \dots , K} |\det A|^{\alpha i_k} \, |c_k| \, \Vert \phi _\delta \Vert _{L^\infty } \\&\gtrsim _{\phi } \delta ^d \Big \Vert \Big (|\det A|^{\alpha i_k} |c_k|\Big )_{k =1}^K \Big \Vert _{\ell ^{\infty }}, \end{aligned}$$

which completes the proof. $\square$

### The case $\alpha \ne 0$

This subsection is devoted to the proof of the following theorem. In particular, it shows that two expansive matrices $A, B \in \textrm{GL}(d, {\mathbb {R}})$ are equivalent whenever $\dot{\textbf{F}}_{p,q}^{\alpha } (A) = \dot{\textbf{F}}_{p,q}^{\alpha } (B)$ and $\alpha \ne 0$. This proves Theorem 5.1 for the case $\alpha \ne 0$.

#### Theorem 5.7

Let $A, B \in \textrm{GL}(d, {\mathbb {R}})$ be expansive, $\alpha , \beta \in {\mathbb {R}}$ and $p_1,p_2, q_1, q_2 \in (0,\infty ]$. If $\dot{\textbf{F}}^{\alpha }_{p_1,q_1}(A) = \dot{\textbf{F}}^{\beta }_{p_2,q_2}(B)$, then the following hold:

(i) $$p_1 = p_2$$,
(ii) $$q_1 = q_2$$,
(iii) $\alpha = \beta$. Furthermore, if $\alpha = \beta \ne 0$, then *A* and *B* are equivalent.

#### Proof

We prove the three assertions separately.

(i) Since $\varphi , \psi \in {\mathcal {S}}({\mathbb {R}}^d)$ are analyzing vectors for *A* resp. *B*, it follows that

5.12 $$\begin{aligned} \bigcup _{i \in {\mathbb {Z}}} (A^*)^{i} Q = \bigcup _{j \in {\mathbb {Z}}} (B^*)^{j} P = {\mathbb {R}}^d {\setminus } \{0\} . \end{aligned}$$

Hence, there exist $i_0, j_0 \in {\mathbb {Z}}$ such that $(A^*)^{i_0} Q \cap (B^*)^{j_0} P \ne \varnothing$. By Corollary 5.4, this implies the existence of some $\delta _0 > 0$ such that for, all $0 < \delta \le \delta _0$,

$$\begin{aligned} |\det A|^{\alpha i_0} \delta ^{d (1-1/p_1)} \asymp |\det B|^{\beta j_0} \delta ^{d (1-1/p_2)}, \end{aligned}$$

with implicit constant independent of $\delta$. In turn, this implies

$$\begin{aligned} \delta ^{1/p_1 - 1/p_2} \asymp 1, \qquad \text {for all} \quad 0 < \delta \le \delta _0, \end{aligned}$$

which is only possible for $p_1 = p_2$.

(ii) Under the assumption $p_1 = p_2 = p$, we show that

5.13 $$\begin{aligned} \Vert c \Vert _{\ell ^{q_1}} \asymp \Vert c \Vert _{\ell ^{q_2}}, \qquad \text {for all} \quad \, K \in {\mathbb {N}}, \; c \in {\mathbb {C}}^K , \end{aligned}$$

where the implied constant is independent of *K* and *c*. This easily implies $q_1 = q_2$.

Let $K \in {\mathbb {N}}$ be arbitrary and let $N \in {\mathbb {N}}$ be as chosen in Eq. (5.4). Recall the identity (5.12) and note that each image set $(A^*)^{i} Q$, $(B^*)^{j} P$ for $i,j \in {\mathbb {Z}}$ is relatively compact and hence bounded. Therefore, it is not hard to see that there exist points $\eta _1,\dots ,\eta _K \in {\mathbb {R}}^d$ and increasing sequences $(i_k)_{k = 1}^K$ and $(j_k)_{k = 1}^K$ in ${\mathbb {Z}}$ satisfying

$$\begin{aligned} |i_k - i_{k'}|> 2N \quad \text {and} \quad |j_k - j_{k'}| > 2N \qquad \text {for } k \ne k', \end{aligned}$$

with

5.14 $$\begin{aligned} \eta _k \in (A^*)^{i_k} Q \cap (B^*)^{j_k} P \quad \text {for all} \quad k = 1,\dots ,K. \end{aligned}$$

Since the sets *Q*, *P* are open, there exists $\delta _1 > 0$ such that

$$\begin{aligned} B_{\delta _1}(\eta _k) \subseteq (A^*)^{i_k} Q \cap (B^*)^{j_k} P \quad \text {for all} \quad k = 1, \dots , K. \end{aligned}$$

Additionally, by continuity of $\phi \in {\mathcal {S}}({\mathbb {R}}^d)$, there exists $\delta _2 > 0$ such that

$$\begin{aligned} |\phi (x)| \ge \frac{1}{2} |\phi (0)|, \qquad x \in \delta _2 ( A^{-i_1}\Omega _A \cup B^{-j_1}\Omega _B). \end{aligned}$$

In combination, this shows that the assumptions of Proposition 5.5 are met for $\dot{\textbf{F}}_{{p},{q_1}}^{{\alpha }}(A;\varphi )$ with $\delta _0:= \min \{\delta _1, \delta _2\}$, $\eta _1,\dots ,\eta _K$ and $(i_k)_{k = 1}^K$, as well as for $\dot{\textbf{F}}_{{p},{q_2}}^{{\beta }}(B;\psi )$ with $(j_k)_{k = 1}^K$ replacing the sequence $(i_k)_{k = 1}^K$.

For showing the claim (5.13), let $c \in {\mathbb {C}}^K$ and $0 < \delta \le \delta _0$ be fixed. Then defining $f^{(\delta )}:= \sum _{k=1}^K |\det A|^{- \alpha i_k} c_k M_{\eta _k} \phi _\delta$ gives

5.15 $$\begin{aligned} \delta ^{d (1 -1/p)}\Vert c \Vert _{\ell ^{q_1}}&\asymp \Vert f^{(\delta )}\Vert _{\dot{\textbf{F}}_{{p},{q_1}}^{{\alpha }}(A)} \nonumber \\&\asymp \Vert f^{(\delta )}\Vert _{\dot{\textbf{F}}_{{p},{q_2}}^{{\beta }}(B)} \nonumber \\&\asymp \delta ^{d (1 -1/p)} \Big \Vert \Big (|\det B|^{\beta j_k} \, |\det A|^{- \alpha i_k}\, |c_k| \Big )_{k =1}^K\Big \Vert _{\ell ^{q_2}}. \end{aligned}$$

Since $(A^*)^{i_k} Q \cap (B^*)^{j_k} P \ne \varnothing$ by Eq. (5.14), it follows by Corollary 5.4 for $p_1 = p_2 = p$ that $|\det A|^{\alpha i_k} \asymp |\det B|^{\beta j_k}$ for $k=1, \dots , K$, with implicit constant independent of $i_k, j_k$. This, together with Eq. (5.15), easily shows the claim (5.13).

(iii) Assuming $p_1 = p_2 = p$, it follows by Corollary 5.4 that there exists $C \ge 1$ such that

5.16 $$\begin{aligned} \frac{1}{C} |\det B|^{\beta j} \le |\det A|^{\alpha i} \le C |\det B|^{\beta j} \quad \text {whenever} \quad (A^*)^i Q \cap (B^*)^j P \ne \varnothing . \end{aligned}$$

We consider the cases $\alpha =0$ or $\beta = 0$, and $\alpha \ne 0 \ne \beta$.

*Case 1* $\alpha = 0$ *or* $\beta = 0$. Suppose first that $\alpha = 0$. As a consequence of Eq. (5.12), for all $j \in {\mathbb {Z}}$, there needs to exist $i \in {\mathbb {Z}}$ such that $(A^*)^i Q \cap (B^*)^j P \ne \varnothing$. Equation (5.16) implies therefore that $|\det B|^{\beta j} \le C$ as $\alpha = 0$. Since this holds for all $j \in {\mathbb {Z}}$, and $|\det B| \ne 0$, it follows that necessarily also $\beta = 0$. If $\beta = 0$, then also $\alpha = 0$ by symmetry.

*Case 2* $\alpha \ne 0 \ne \beta$. Suppose that $\alpha \ne 0 \ne \beta$. Then, by Eq. (5.16), the assumptions of Lemma 2.5 are satisfied for $\big ( (A^*)^i Q \big )_{i \in {\mathbb {Z}}}$ and $\big ( (B^*)^j P \big )_{j \in {\mathbb {Z}}}$. Hence, there exists $M \in {\mathbb {N}}$ such that with $J_i,I_j$ as defined in Eq. (5.3), we have

$$\begin{aligned} J_i \subseteq \Big \{ j \in {\mathbb {Z}}:\Big |j - \Big \lfloor \frac{\alpha }{\beta } \, c \, i \Big \rfloor \Big | \le M \Big \}, \quad \text {and} \quad I_j \subseteq \Big \{ i \in {\mathbb {Z}}:\Big |i -\Big \lfloor \frac{\beta }{\alpha } \, \frac{1}{c} \, j \Big \rfloor \Big | \le M \Big \}, \end{aligned}$$

where $c = c(A, B):= \ln |\det A| / \ln |\det B|$. In particular, this implies that

$$\begin{aligned} \sup _{j \in {\mathbb {Z}}} |I_j| + \sup _{i \in {\mathbb {Z}}} |J_i| < \infty . \end{aligned}$$

Therefore, an application of Lemma 2.3 implies that $A^*$ and $B^*$ are equivalent, and hence so are *A* and *B* by Corollary 2.2.

It remains to show that $\alpha = \beta$. To see this, note that, for all $j \in {\mathbb {Z}}$, it holds that

$$\begin{aligned} |\det B|^j&\lesssim _{P} \textrm{m} \left( {(B^*)^j P}\right) \le \textrm{m}\Big (\bigcup _{i \in I_j} (A^*)^i Q \Big ) \le \sum _{i \in I_j} \textrm{m} \left( {(A^*)^i Q}\right) \\&\lesssim _{Q} \sum _{k = -M}^M |\det A|^{\lfloor \frac{\beta }{\alpha } \, \frac{1}{c} \, j \rfloor +k} \lesssim _{M,A} |\det A|^{ \frac{\beta }{\alpha } \frac{\ln (|\det B|)}{\ln (|\det A|)} j} = |\det B|^{ \frac{\beta }{\alpha }j} . \end{aligned}$$

Since $|\det B| \ne 0$, this is only possible for $\frac{\beta }{\alpha } = 1$, and hence $\alpha = \beta$ as claimed. $\square$

### The case $\alpha = 0$ and $p < \infty$

In this section, we prove the following theorem, showing that if two Triebel–Lizorkin spaces coincide and the matrices are *not* equivalent, then necessarily $q = 2$. The only shortcoming of this theorem is that it only applies when $p < \infty$. We will deal with the case $p = \infty$ in the following subsection.

#### Theorem 5.8

Let $A,B \in \textrm{GL}(d,{\mathbb {R}})$ be expansive, $p \in (0,\infty )$ and $q \in (0,\infty ]$. Suppose that

5.17 $$\begin{aligned} \Vert f \Vert _{\dot{\textbf{F}}^{0}_{p,q}(A)} \asymp \Vert f \Vert _{\dot{\textbf{F}}^{0}_{p,q}(B)}, \quad \text {for all} \quad f \in {\mathcal {F}}^{-1} (C_c^{\infty } ({\mathbb {R}}^d {\setminus } \{0\})). \end{aligned}$$

If *A* and *B* are not equivalent, then $q = 2$.

In particular, if $\dot{\textbf{F}}^{0}_{p,q}(A) = \dot{\textbf{F}}^{0}_{p,q}(B)$ and *A* and *B* are not equivalent, then $q=2$.

The following observation will be key in proving Theorem 5.8. It provides a condition under which the hypotheses of Proposition 5.5 are satisfied for $\dot{\textbf{F}}^{\alpha }_{p,q}(A)$.

#### Lemma 5.9

Let $A, B \in \textrm{GL}(d, {\mathbb {R}})$ be expansive and suppose that $\sup _{j \in {\mathbb {Z}}} |I_j| = \infty$, with $I_j$ as defined in Eq. (5.3).

Then, for every $K \in {\mathbb {N}}$, there exist $\delta _0 > 0$, $j_0 \in {\mathbb {Z}}$, points $\eta _1, \dots \eta _K \in {\mathbb {R}}^d$, and a (strictly) increasing sequence $i_1, \dots , i_K \in {\mathbb {Z}}$ with $|i_k - i_{k'}| >2N$ for $k \ne k'$, where $N \in {\mathbb {N}}$ as in (5.4), such that the following assertions hold:

(i) $$B_{\delta _0}(\eta _k) \subseteq (A^*)^{i_k}Q \cap (B^*)^{j_0} P$$ for all $$k = 1, \dots , K$$;
(ii) $|\phi (x)| \ge \frac{1}{2}|\phi (0)|$ for all $x \in \delta _0 A^{-i_1}\Omega _A$.

In particular, the assumptions (a) and (b) of Proposition 5.5 are satisfied for $\dot{\textbf{F}}^{\alpha }_{p,q}(A)$.

#### Proof

Let $K \in {\mathbb {N}}$ be arbitrary. Then, since $\sup _{j \in {\mathbb {Z}}} |I_j| = \infty$, there exists $j_0 \in {\mathbb {Z}}$ such that $|I_{j_0}| \ge (2 N + 1) K$. Define ${\mathbb {Z}}_n:= n + (2 N + 1) \, {\mathbb {Z}}$ for $n = 0,\dots ,2N$. Since $I_{j_0} = \bigcup _{n = 0}^{2N} (I_{j_0} \cap {\mathbb {Z}}_n)$, there needs to be at least one $n_0 \in \{0, \dots , 2N\}$ such that $|I_{j_0} \cap {\mathbb {Z}}_{n_0}| \ge K$. Hence, we can choose a strictly increasing sequence $i_1, \dots , i_K \in I_{j_0} \cap {\mathbb {Z}}_{n_0}$, which in particular implies that $|i_k - i_{k'}| \ge 2N+1$ for $k \ne k'$. Since $(A^*)^{i_k}Q \cap (B^*)^{j_0}P \ne \varnothing$ is open for all $k=1, \dots , N$, there exist $\eta _1, \dots , \eta _K$ and a constant $\delta _1 > 0$ such that

$$\begin{aligned} B_{\delta _1}(\eta _k) \subseteq (A^*)^{i_k}Q \cap (B^*)^{j_0} P \qquad \text {for all} \quad k=1, \dots , K. \end{aligned}$$

Finally, continuity of $\phi \in {\mathcal {S}}({\mathbb {R}}^d)$ implies (because of $|\phi (0)| = \phi (0) = \int _{{\mathbb {R}}^d} \widehat{\phi (\xi )} \, d \xi > 0$) the existence of $\delta _2 > 0$ such that $|\phi (x)| \ge \frac{1}{2}|\phi (0)|$ for all $x \in \delta _2 A^{-i_1}\Omega _A$, which completes the proof by setting $\delta _0:= \min \{\delta _1, \delta _2\}$. $\square$

Another key ingredient used in the proof of Theorem 5.8 is Khintchine’s inequality, see, e.g., [27, Proposition 4.5]. We include its statement for the convenience of the reader.

#### Lemma 5.10

(Khintchine) Let $\theta = (\theta _1, \dots , \theta _K)$ be a random vector with $\theta \sim U(\{ \pm 1 \}^K)$ (i.e., ${\mathbb {P}}(\theta = \eta ) = \frac{1}{2^K}$ for every $\eta \in \{ \pm 1 \}^K$). For any $p \in (0, \infty )$, denoting the expectation with respect to $\theta$ by ${\mathbb {E}}_{\theta }$, it holds that

$$\begin{aligned} {\mathbb {E}}_{\theta } \bigg |\sum _{k=1}^K \theta _k \, a_k\bigg |^p \asymp \bigg (\sum _{k=1}^{K} |a_k|^2\bigg )^{p/2} \qquad \text {for all} \quad (a_k)_{k = 1}^K \in {\mathbb {C}}^K, \end{aligned}$$

where the implied constant only depends on *p*.

We will now provide the proof of Theorem 5.8.

#### Proof of Theorem 5.8

If *A* and *B* are not equivalent, then neither are $A^*$ and $B^*$ (cf. Corollary 2.2). Hence, an application of Lemma 2.3 implies for $\big ((A^*)^i Q\big )_{i \in {\mathbb {Z}}}$ and $\big ((B^*)^j P\big )_{j \in {\mathbb {Z}}}$ that

$$\begin{aligned} \sup _{i \in {\mathbb {Z}}} |J_i| + \sup _{j \in {\mathbb {Z}}} |I_j| = \infty . \end{aligned}$$

By exchanging the roles of *A* and *B* if necessary, it may be assumed that $\sup _{j \in {\mathbb {Z}}} |I_j| = \infty$, so that the assumption of Lemma 5.9 is satisfied. Using Lemma 5.9, it will be shown that

5.18 $$\begin{aligned} \Vert c \Vert _{\ell ^q} \asymp \Vert c \Vert _{\ell ^2 } \qquad \text {for all} \quad \, K \in {\mathbb {N}}\text { and } c \in {\mathbb {C}}^K, \end{aligned}$$

where the implied constant is independent of *K* and *c*. This easily implies $q = 2$.

For showing (5.18), let $K \in {\mathbb {N}}$ and $c \in {\mathbb {C}}^K$ be arbitrary. Then an application of Lemma 5.9 yields some $j_0 \in {\mathbb {Z}}$, points $\eta _1, \dots , \eta _K \in {\mathbb {R}}^d$, a strictly increasing sequence $i_1, \dots , i_K \in {\mathbb {Z}}$, and $\delta _0 > 0$ such that $B_{\delta _0}(\eta _k) \subseteq (B^*)^{j_0} P$ for all $k \in \{ 1,\dots ,K \}$ and such that the assumptions of Proposition 5.5 are satisfied. Proposition 5.5 thus implies for fixed but arbitrary $0 < \delta \le \delta _0$, and any $\theta \in \{ \pm 1 \}^K$ that the function $f_{\theta , \delta }: = \sum _{k = 1}^K \theta _k \, c_k M_{\eta _k}\phi _\delta$ satisfies $\Vert f_{\theta , \delta } \Vert _{\dot{\textbf{F}}^{0}_{p,q}(A)} \asymp \delta ^{d (1 - \frac{1}{p})} \Vert c \Vert _{\ell ^q}$. On the other hand, it holds $\textrm{supp}\,\widehat{f_{\theta , \delta }} \subseteq (B^*)^{j_0} P$ for all $0 < \delta \le \delta _0$, and thus Proposition 5.3 is applicable for $\dot{\textbf{F}}^{0}_{p,q}(B)$. Consequently, Eq. (5.17) implies that

5.19 $$\begin{aligned} \delta ^{d (1 -1/p)} \Vert c \Vert _{\ell ^q } \asymp \Vert f_{\theta , \delta } \Vert _{\dot{\textbf{F}}^{0}_{p,q}(A)} \asymp \Vert f_{\theta , \delta } \Vert _{\dot{\textbf{F}}^{0}_{p,q}(B)} \asymp \Vert f_{\theta , \delta } \Vert _{L^p} \qquad \text {for all} \quad \theta \in \{ \pm 1 \}^K. \end{aligned}$$

Using Khintchine’s inequality (Lemma 5.10), we see that if we take $\theta \sim U(\{ \pm 1 \}^K)$ as a random vector, then

$$\begin{aligned} {\mathbb {E}}_{\theta } \Vert f_{\theta , \delta } \Vert _{L^p}^p&= {\mathbb {E}}_{\theta } \int _{{\mathbb {R}}^d} \bigg | \sum _{k=1}^{K} \theta _k \, c_k \, e^{2 \pi i \eta _k \cdot x } \bigg |^p |\phi _\delta (x)|^p \, d x \\&= \int _{{\mathbb {R}}^d} |\phi _\delta (x)|^p \, {\mathbb {E}}_{\theta } \bigg | \sum _{k=1}^{K} \theta _k \, c_k \, e^{2 \pi i \eta _k \cdot x } \bigg |^p \, d x \\&\asymp _p \int _{{\mathbb {R}}^d} |\phi _\delta (x)|^p \bigg ( \sum _{k=1}^{K} |c_k \,e^{2 \pi i \eta _k \cdot x }|^2 \bigg )^{p/2} \, d x \asymp _{p,\phi } \delta ^{d (p-1)} \Vert c \Vert _{\ell ^2}^p . \end{aligned}$$

In combination with (5.19), this easily implies that Eq. (5.18) holds. $\square$

The finer analysis in the case where $\alpha = 0$ and $q = 2$ can be performed by using that $\dot{\textbf{F}}^{0}_{p,2}(A)$ coincides with the anisotropic Hardy space $H^p (A)$ and using the classification results of [2, Section 10]. The details are as follows:

#### Theorem 5.11

Let $A, B \in \textrm{GL}(d, {\mathbb {R}})$ be expansive and $p \in (0,\infty )$. If $\dot{\textbf{F}}^{0}_{p,2}(A) = \dot{\textbf{F}}^{0}_{p,2}(B)$, then at least one of the following cases holds:

(i) *A* and *B* are equivalent, or
(ii) $$p \in (1, \infty )$$.

#### Proof

Let $p \in (0,\infty )$ and denote by $H^p(A)$ the anisotropic Hardy space introduced in [2]. By [4, Theorem 7.1], it follows that $\dot{\textbf{F}}^{0}_{p,2}(A) = H^p (A)$. Hence, if $\dot{\textbf{F}}^{0}_{p,2}(A) = \dot{\textbf{F}}^{0}_{p,2}(B)$, then $H^p (A) = H^p (B)$.

If $p \in (0, 1]$, then by [2, Theorem 10.5] (see also [7, Theorem 2.3] for a corrected statement), the identity $H^p (A) = H^p (B)$ implies that *A* and *B* are equivalent. Thus, (i) holds. $\square$

A combination of Theorems 5.8 and 5.11 yields the following:

#### Corollary 5.12

Let $A,B \in \textrm{GL}(d,{\mathbb {R}})$ be expansive, $p \in (0,\infty )$ and $q \in (0,\infty ]$. Suppose that $\dot{\textbf{F}}^{0}_{p,q}(A) = \dot{\textbf{F}}^{0}_{p,q}(B)$. Then at least one of the following cases holds:

(i) *A* and *B* are equivalent;
(ii) $$q = 2$$ and $$p \in (1,\infty )$$.

### The case $\alpha = 0$ and $p = \infty$

This section provides the following theorem, which finishes the necessary conditions of Theorem 5.1.

#### Theorem 5.13

Let $A,B \in \textrm{GL}(d, {\mathbb {R}})$ be expansive and $q \in (0, \infty ]$. If $\dot{\textbf{F}}^{0}_{\infty ,q} (A) = \dot{\textbf{F}}^0_{\infty ,q}(B)$, then *A* and *B* are equivalent.

The following lemma will reduce the proof of Theorem 5.13 to the case $q \ge 1$.

#### Lemma 5.14

Let $A,B \in \textrm{GL}(d, {\mathbb {R}})$ be expansive and $q \in (0, \infty ]$. If $\dot{\textbf{F}}^{0}_{\infty ,q} (A) = \dot{\textbf{F}}^0_{\infty ,q}(B)$ and the matrices *A* and *B* are not equivalent, then $q \ge 1$.

#### Proof

The claim is trivial for $q = \infty$; therefore, we can assume that $q < \infty$. Since *A* and *B* are not equivalent, Corollary 2.2 and Lemma 2.3 again imply for the covers $\big ((A^*)^i Q\big )_{i \in {\mathbb {Z}}}$ and $\big ((B^*)^j P\big )_{j \in {\mathbb {Z}}}$ that

$$\begin{aligned} \sup _{i \in {\mathbb {Z}}} |J_i| + \sup _{j \in {\mathbb {Z}}} |I_j| = \infty , \end{aligned}$$

where we may assume $\sup _{j \in {\mathbb {Z}}} |I_j| = \infty$ by interchanging *A* and *B* if necessary.

For $K \in {\mathbb {N}}$ arbitrary, we now invoke Lemma 5.9 to obtain $j_0 \in {\mathbb {Z}}$, $\eta _1, \dots , \eta _K \in {\mathbb {R}}^d$, a strictly increasing sequence $i_1, \dots , i_K \in {\mathbb {Z}}$, and some $\delta _0 > 0$ such that the assumptions of Proposition 5.5 are satisfied and such that $B_{\delta _0}(\eta _k) \subseteq (B^*)^{j_0} P$ for all $k \in \{ 1,\dots ,K \}$. Proposition 5.5 thus implies for any $0 < \delta \le \delta _0$ that each of the functions

$$\begin{aligned} f_{c, \delta } := \sum _{k=1}^{K} c_k M_{\eta _k} \phi _\delta , \qquad c \in {\mathbb {C}}^K, \end{aligned}$$

satisfies $\Vert f_{c, \delta } \Vert _{\dot{\textbf{F}}^{0}_{\infty ,q} (A)} \asymp \delta ^d \, \Vert c \Vert _{\ell ^q}$. Since $\textrm{supp}\,\widehat{f_{c, \delta }} \subseteq (B^*)^{j_0}P$, Proposition 5.3 is applicable for $\dot{\textbf{F}}^{0}_{\infty ,q} (B)$. Consequently, and recalling (5.2), we see that

$$\begin{aligned} \delta ^d \, \Vert c\Vert _{\ell ^q} \asymp \Vert f_{c, \delta } \Vert _{\dot{\textbf{F}}^0_{\infty ,q} (A)} \asymp \Vert f_{c, \delta } \Vert _{\dot{\textbf{F}}^0_{\infty ,q} (B)} \asymp \Vert f_{c, \delta } \Vert _{L^\infty } \le \Vert c \Vert _{\ell ^1} \, \Vert \phi _\delta \Vert _{L^\infty } \lesssim \delta ^d \, \Vert c \Vert _{\ell ^1}, \end{aligned}$$

which can only hold for $q \ge 1$. $\square$

By duality, we now provide a proof of Theorem 5.13.

#### Proof of Theorem 5.13

Arguing by contradiction, we assume that *A* and *B* are not equivalent. Then Lemma 5.14 implies that $q \ge 1$.

First, suppose that $q \in (1, \infty ]$, so that its conjugate exponent $q'$ satisfies $q' \in [1,\infty )$. Then [5, Theorem 4.8] shows that $\dot{\textbf{F}}^0_{\infty ,q} (A)$ is the dual space of $\dot{\textbf{F}}^0_{1,q'}(A)$ (with equivalent norms). Likewise, it follows that $\dot{\textbf{F}}^0_{\infty ,q}(B)$ is the dual space of $\dot{\textbf{F}}^0_{1,q'}(B)$ (with equivalent norms). By the first part of Lemma 5.2, we have for $f\in [\dot{\textbf{F}}^0_{1,q'}(A)]' = \dot{\textbf{F}}^0_{\infty ,q}(A) = \dot{\textbf{F}}^0_{\infty ,q}(B) = [\dot{\textbf{F}}^0_{1,q'}(B)]'$ that

$$\begin{aligned} \Vert f \Vert _{[\dot{\textbf{F}}^0_{1,q'} (A)]'} \asymp \Vert f \Vert _{\dot{\textbf{F}}^0_{\infty ,q} (A)} \asymp \Vert f \Vert _{\dot{\textbf{F}}^0_{\infty ,q} (B)} \asymp \Vert f \Vert _{[\dot{\textbf{F}}^0_{1,q'} (B)]'}. \end{aligned}$$

Therefore, by the usual dual characterization of the norm, it holds that

$$\begin{aligned} \Vert g \Vert _{\dot{\textbf{F}}^0_{1,q'}(A)} = \sup _{\begin{array}{c} f \in [\dot{\textbf{F}}^0_{1,q'}(A)]'\\ \Vert f \Vert _{[\dot{\textbf{F}}^0_{1,q'}(A)]'} \le 1 \end{array}} |\langle f, g \rangle | \asymp \sup _{\begin{array}{c} f \in [\dot{\textbf{F}}^0_{1,q'}(B)]'\\ \Vert f \Vert _{[\dot{\textbf{F}}^0_{1,q'}(B)]'} \le 1 \end{array}} |\langle f, g \rangle | = \Vert g \Vert _{\dot{\textbf{F}}^0_{1,q'}(B)}, \quad g \in {\mathcal {S}}_{0} ({\mathbb {R}}^d). \end{aligned}$$

Second, if $q = 1$, then it follows directly from Proposition B.6 that

$$\begin{aligned} \Vert g \Vert _{\dot{\textbf{F}}^{0}_{1,\infty }(A)} \asymp \sup _{\begin{array}{c} f \in \dot{\textbf{F}}^0_{\infty , 1}(A) \\ \Vert f \Vert _{\dot{\textbf{F}}^0_{\infty , 1}(A) } \le 1 \end{array} } |\langle f, g \rangle | \asymp \sup _{\begin{array}{c} f \in \dot{\textbf{F}}^0_{\infty , 1}(B) \\ \Vert f \Vert _{\dot{\textbf{F}}^0_{\infty , 1}(B) } \le 1 \end{array} } |\langle f, g \rangle | \asymp \Vert g \Vert _{\dot{\textbf{F}}^{0}_{1,\infty }(B)}, \quad g \in {\mathcal {S}}_{0} ({\mathbb {R}}^d). \end{aligned}$$

In combination, for any $q \in [1, \infty ]$, this yields $\Vert g \Vert _{\dot{\textbf{F}}^{0}_{1,q'}(A)} \asymp \Vert g \Vert _{\dot{\textbf{F}}^{0}_{1,q'}(B)}$ for all $g \in {\mathcal {S}}_{0} ({\mathbb {R}}^d)$. Since *A* and *B* are not equivalent, an application of Theorem 5.8 shows that $q' = 2$ and hence $q = 2$. But for $p = 1$, $q = 2$, the above norm equivalence holds on a common dense subset, hence $\dot{\textbf{F}}^{0}_{1,2}(A) = \dot{\textbf{F}}^{0}_{1,2}(B)$ by the second part of Lemma 5.2. Now Theorem 5.11 implies that *A* and *B* need to be equivalent, a contradiction. $\square$

## Data Availability

Data sharing not applicable to this article as no datasets were generated or analyzed during the current study.

## Appendix A: Miscellaneous results

This section contains two results used in the proofs of the main theorems. The most important such result is the following convolution relation, which is [24, Proposition in Section 1.5.1] with the implied constant written out explicitly. A proof can also be found in [26, Theorem 3.4].

### Proposition A.1

([24]) Let $K_1, K_2 \subseteq {\mathbb {R}}^d$ be compact and $p \in (0,1]$. If $f, \psi \in {\mathcal {S}}({\mathbb {R}}^d)$ satisfy $\textrm{supp}\,\widehat{\psi } \subseteq K_1$ and $\textrm{supp}\,\widehat{f} \subseteq K_2$, then the following quasi-norm estimate holds:

$$\begin{aligned} \Vert f *\psi \Vert _{L^p} \le [\textrm{m} \left( {K_1 - K_2}\right) ]^{\frac{1}{p} -1} \Vert f \Vert _{L^p} \Vert \psi \Vert _{L^p}, \end{aligned}$$

where $\textrm{m} \left( {K_1 - K_2}\right)$ denotes the Lebesgue measure of $K_1 - K_2:= \{u - v: u \in K_1, v \in K_2 \}$.

### Corollary A.2

Let $A \in \textrm{GL}(d, {\mathbb {R}})$ be expansive, let $K \subseteq {\mathbb {R}}^d$ be compact, and let $N \in {\mathbb {N}}$ and $p \in (0,1)$. Then there exists a constant $C = C(A,K,N,p) > 0$ with the following property:

If $f,g \in {\mathcal {S}}({\mathbb {R}}^d)$ satisfy $\textrm{supp}\,\widehat{f}, \textrm{supp}\,\widehat{g} \subseteq \bigcup _{\ell =-N}^N (A^*)^{i + \ell } K$ for some $i \in {\mathbb {Z}}$, then

$$\begin{aligned} \Vert f *g \Vert _{L^p} \le C |\det A|^{i \left( \frac{1}{p} - 1\right) } \Vert f \Vert _{L^p} \Vert g \Vert _{L^p}. \end{aligned}$$

### Proof

By compactness of $K \subseteq {\mathbb {R}}^d$, there exists $R = R(A,K,N) > 0$ such that

$$\begin{aligned} \bigcup _{\ell =-N}^N (A^*)^{\ell } K \subseteq \overline{B}_R(0). \end{aligned}$$

Setting $K_1:= K_2:= (A^*)^i \overline{B}_R (0)$, it follows that $\textrm{supp}\,\widehat{f} \subseteq K_1$, $\textrm{supp}\,\widehat{g} \subseteq K_2$, and

$$\begin{aligned} \textrm{m} \left( {K_1 - K_2}\right) \le \textrm{m} \left( {(A^*)^i \overline{B}_{2R}(0)}\right) = |\det A|^i \cdot \textrm{m} \left( {\overline{B}_{2R}(0)}\right) . \end{aligned}$$

Hence, an application of Proposition A.1 easily yields the claim. $\square$

The second result is the following technical estimate.

### Lemma A.3

Let $A \in \textrm{GL}(d,{\mathbb {R}})$ be expansive, let $M > 0$, $N \in {\mathbb {N}}$, and $Q \subseteq {\mathbb {R}}^d$ be bounded. Further, let $\varphi ,\phi$ as in Sect. 5.1. Then there exists a constant $C = C(d,M,N,Q,\phi ,\varphi ,A) > 0$ with the following property:

If $i,\ell \in {\mathbb {Z}}$ and $\delta > 0$ are such that $|i - \ell | \le N$ and $B_\delta (\eta ) \subseteq (A^*)^\ell Q$ for some $\eta \in {\mathbb {R}}^d$, then

$$\begin{aligned} \big ( |\phi _\delta | *|\varphi _i| \big ) (x) \le C \delta ^d (1 + |\delta x|)^{-M} \end{aligned}$$

holds for all $x \in {\mathbb {R}}^d$.

### Proof

Let $R = R(Q) > 0$ be such that $Q \subseteq B_R (0)$. Then

$$\begin{aligned} B_\delta (0) \subseteq B_\delta (\eta ) - B_\delta (\eta ) \subseteq (A^*)^\ell Q - (A^*)^\ell Q \subseteq (A^*)^\ell B_{2 R} (0), \end{aligned}$$

and thus $(A^*)^{-\ell } B_1(0) \subseteq B_{2 R / \delta } (0)$, so that $\Vert (A^*)^{-\ell } \Vert \le 2 R / \delta$. Therefore,

$$\begin{aligned} \Vert A^{-i} \Vert = \Vert (A^*)^{-i} \Vert = \Vert (A^*)^{-\ell } (A^*)^{\ell - i} \Vert \lesssim _{A,N} \Vert (A^*)^{-\ell } \Vert \le 2 R / \delta , \end{aligned}$$

where it is used that $|i - \ell | \le N$. Thus, given any $y \in {\mathbb {R}}^d$, it follows that $|\delta y| \le C_1 \, |A^i y|$ for a certain constant $C_1 = C_1(A,N,Q) \ge 1$. This implies, for arbitrary $x,y \in {\mathbb {R}}^d$, that

$$\begin{aligned} 1 + |\delta x| \le (1 + |\delta (x-y)|) (C_1 + |\delta y|) \le C_1 (1 + |\delta (x-y)|) (1 + |A^i y|). \end{aligned}$$

By rearranging, this shows $(1 + |\delta (x-y)|)^{-M} \le C_1^M (1 + |\delta x|)^{-M} (1 + |A^i y|)^M$ for all $x, y \in {\mathbb {R}}^d$.

Next, since $\phi ,\varphi \in {\mathcal {S}}({\mathbb {R}}^d)$, there exists $C_2 = C_2(\phi ,\varphi ,M,d) > 0$ such that

$$\begin{aligned} |\phi (x)| \le C_2 (1 + |x|)^{-M} \qquad \text {and} \qquad |\varphi (x)| \le C_2 (1 + |x|)^{-(M + d + 1)} \end{aligned}$$

for all $x \in {\mathbb {R}}^d$. Hence,

$$\begin{aligned} \big ( |\phi _\delta | *|\varphi _i| \big ) (x)&\le \delta ^d \, |\det A|^i \int _{{\mathbb {R}}^d} |\phi (\delta (x-y))| |\varphi (A^i y)| \, d y \\&\le C_2^2 \, \delta ^d \, |\det A^i| \int _{{\mathbb {R}}^d} (1 + |\delta (x-y)|)^{-M} (1 + |A^i y|)^{-(M + d + 1)} \, d y \\&\le C_1^M C_2^2 \, \delta ^d \, (1 + |\delta x|)^{-M} \int _{{\mathbb {R}}^d} |\det A^i| (1 + |A^i y|)^{-(d+1)} \, d y \\&= C_1^M \, C_2^2 \, \delta ^d (1 + |\delta x|)^{-M} \, \int _{{\mathbb {R}}^d} (1 + |z|)^{-(d+1)} \, d z . \end{aligned}$$

This easily implies the claim of the lemma. $\square$

## Appendix B: Equivalent norm for $\dot{\textbf{F}}^{0}_{1,\infty }(A)$

This section provides a dual characterization for the norm of $\dot{\textbf{F}}^{0}_{1,\infty }(A)$, which is used in the proof of Theorem 5.13. Its proof hinges on associated Triebel–Lizorkin sequence spaces for which we recall the basic objects first.

Let $A \in \textrm{GL}(d, {\mathbb {R}})$ be an expansive matrix and let ${\mathcal {D}}_A$ be the collection of all *dilated cubes*

$$\begin{aligned} {\mathcal {D}}_A = \big \{ D = A^i ([0,1]^d + k): i \in {\mathbb {Z}}, k \in {\mathbb {Z}}^d \big \} \end{aligned}$$

associated to *A*. The *scale* of a dilated cube $D = A^i ([0,1]^d + k) \in {\mathcal {D}}_A$ is defined as $\textrm{scale} (D) = i$; alternatively, $\textrm{scale}(D) = \log _{|\det A|} \textrm{m}(D)$. The *tent* over $D \in {\mathcal {D}}_A$ is defined as

$$\begin{aligned} {\mathcal {T}}(D):= \big \{ D' \in {\mathcal {D}}_A: \textrm{m}(D' \cap D) > 0 \quad \text {and} \quad \textrm{scale}(D') \le \textrm{scale}(D) \big \}. \end{aligned}$$

The following lemma provides a convenient cover for the union of elements of a tent and will be used in two proofs below.

### Lemma B.1

There exists $N = N(A,d) \in {\mathbb {N}}$ such that for all $D \in {\mathcal {D}}_A$, we have

$$\begin{aligned} \bigcup _{D' \in {\mathcal {T}}(D)} D' \subseteq \bigcup _{\begin{array}{c} n \in {\mathbb {Z}}^d \\ |n| \le N \end{array}} (D + A^{\textrm{scale}(D)}n). \end{aligned}$$

### Proof

First, let $D' = A^i([0,1]^d+k) \in {\mathcal {D}}_A$ with $i \le 0$. Then

B.1 $$\begin{aligned} \textrm{diam} (D') := \max _{z_1, z_2 \in D'} |z_1 - z_2| = \max _{x_1, x_2 \in [0,1]^d} |A^i(x_1 - x_2)| \le C \lambda _-^i \sqrt{d}, \end{aligned}$$

where the inequality used that $| A^i x | \le C \lambda _-^i | x|$ for all $x \in {\mathbb {R}}^d$, see, e.g., [2, Equations (2.1) and (2.2)]. Since $\lambda _- > 1$, we can choose $R > 0$ such that $R > C \lambda _-^i \sqrt{d}$ for all $i \le 0$. Then, for arbitrary $D' \in {\mathcal {T}}([0,1]^d)$, it follows that $D' \cap [0,1]^d \ne \varnothing$, and hence $\textrm{dist} (x, [0,1]^d) < R$ for all $x \in D'$, so that $D' \subseteq [0,1]^d + B_R (0)$. Therefore,

B.2 $$\begin{aligned} \bigcup _{D' \in {\mathcal {T}}([0,1]^d)} D' \subseteq [0,1]^d + B_R (0) \subseteq \bigcup _{\begin{array}{c} n \in {\mathbb {Z}}^d \\ |n| \le N \end{array}} ([0,1]^d + n) \end{aligned}$$

for some $$N = N(A,d) > 0$$.

Second, if $D' = A^i ([0,1]^d + k) \in {\mathcal {T}}([0,1]^d + \ell )$ for some $\ell \in {\mathbb {Z}}^d$, then $D' \cap ([0,1]^d + \ell ) \ne \varnothing$ implies that $\textrm{dist}(x, [0,1]^d + \ell ) \le \textrm{diam}(D') < R$ for all $x \in D'$ by the arguments following (B.1). Therefore, by Eq. (B.2),

B.3 $$\begin{aligned} \bigcup _{D' \in {\mathcal {T}}([0,1]^d + \ell )} D' \subseteq [0,1]^d + B_R (0) + \ell \subseteq \bigcup _{\begin{array}{c} n \in {\mathbb {Z}}^d \\ |n| \le N \end{array}} ([0,1]^d + \ell + n). \end{aligned}$$

At last, let $D = A^j ([0,1]^d + \ell ) \in {\mathcal {D}}_A$ be arbitrary. Then $D' = A^i([0,1]^d + k) \in {\mathcal {T}}(D)$ means $\textrm{m}(D' \cap D) > 0$ and $i \le j$ by definition of ${\mathcal {T}}(D)$. This is clearly equivalent to

$$\begin{aligned}{} & {} |\det A|^j \textrm{m}\bigl ( A^{i - j} ([0,1]^d + k) \cap [0,1^d] + \ell \bigr ) \\{} & {} \quad = \textrm{m}\bigl ( A^j (A^{i-j} [0,1]^d + k) \cap A^j([0,1]^d + \ell )\bigr ) > 0 \end{aligned}$$

and $i - j \le 0$. Thus, $D' = A^i ([0,1]^d + k) \in {\mathcal {T}}(D)$ if and only if

$$\begin{aligned} A^{-j} D' = A^{i-j} ([0,1]^d + k) \in {\mathcal {T}}([0,1]^d + \ell ). \end{aligned}$$

Using Eq. (B.3), it follows therefore that

$$\begin{aligned} \bigcup _{D' \in {\mathcal {T}}(D)} D' \subseteq A^j \bigg ( \bigcup _{\begin{array}{c} n \in {\mathbb {Z}}^d \\ |n| \le N \end{array}} ([0,1]^d + \ell + n) \bigg ) = \bigcup _{\begin{array}{c} n \in {\mathbb {Z}}^d \\ |n| \le N \end{array}} (D + A^j n), \end{aligned}$$

as required. $$\square $$

The Triebel–Lizorkin sequence spaces $\dot{\textbf{f}}^0_{1, \infty } (A)$ and $\dot{\textbf{f}}^0_{\infty , 1}(A)$ are defined as the collections of all complex-valued sequences $c = (c_D)_{D \in {\mathcal {D}}_A}$ satisfying

$$\begin{aligned} \Vert c \Vert _{\dot{\textbf{f}}^0_{1, \infty } (A)}:= \int _{{\mathbb {R}}^d} \sup _{D \in {\mathcal {D}}_A} \textrm{m}(D)^{-1/2} |c_D| \mathbbm {1}_D (x) \; dx < \infty \end{aligned}$$

and

B.4 $$\begin{aligned} \Vert c \Vert _{\dot{\textbf{f}}^0_{\infty , 1}(A)} := \sup _{D' \in {\mathcal {D}}_A} \frac{1}{\textrm{m}(D')} \int _{D'} \sum _{\begin{array}{c} D \in {\mathcal {D}}_A \\ \textrm{scale}(D) \le \textrm{scale}(D') \end{array}} \textrm{m}(D)^{-1/2} |c_D| \mathbbm {1}_D (x) \; dx < \infty , \end{aligned}$$

respectively.

The following simple characterization of $\textbf{f}^0_{\infty , 1} (A)$ will be used below. This equivalence is already claimed in [4, Remark 3.5], but a short proof is included for the sake of completeness.

### Lemma B.2

For all complex-valued sequences $c = (c_D)_{D \in {\mathcal {D}}_A}$,

B.5 $$\begin{aligned} \Vert c \Vert _{\dot{\textbf{f}}^0_{\infty , 1}(A)} \asymp \sup _{D' \in {\mathcal {D}}_A} \frac{1}{\textrm{m}(D')} \sum _{D \in {\mathcal {T}}(D')} \textrm{m}(D)^{1/2} |c_D|, \end{aligned}$$

where ${\mathcal {T}}(D')$ denotes the tent over $D' \in {\mathcal {D}}_A$.

### Proof

First, note that interchanging the sum and integral in Eq. (B.4) yields that

B.6 $$\begin{aligned} \Vert c \Vert _{\dot{\textbf{f}}^0_{\infty , 1}(A)} = \sup _{D' \in {\mathcal {D}}_A} \frac{1}{\textrm{m}(D')} \sum _{\begin{array}{c} D \in {\mathcal {D}}_A \\ \textrm{scale}(D) \le \textrm{scale}(D') \end{array}} \textrm{m}(D)^{-1/2} |c_D| \, \textrm{m}(D \cap D'), \end{aligned}$$

which easily implies the claimed inequality $\lesssim$ in Eq. (B.5).

For the reverse inequality, let $D' = A^j ([0,1]^d + \ell ) \in {\mathcal {D}}_A$ be arbitrary. Then an application of Lemma B.1 yields $N = N(A,d) \in {\mathbb {N}}$ such that

$$\begin{aligned} T_{D'}&:= \frac{1}{\textrm{m}(D')} \sum _{D \in {\mathcal {T}}(D')} |c_D| \textrm{m}(D)^{-1/2} \textrm{m}(D) \\&= \frac{1}{\textrm{m}(D')} \sum _{D \in {\mathcal {T}}(D')} |c_D| \textrm{m}(D)^{-1/2} \sum _{\begin{array}{c} n \in {\mathbb {Z}}^d \\ |n| \le N \end{array}} \textrm{m}\bigl (D \cap A^j ([0,1]^d + \ell + n)\bigr ) \\&\le \frac{1}{\textrm{m}(D')} \sum _{\begin{array}{c} D \in {\mathcal {D}}_A \\ \textrm{scale}(D) \le j \end{array}} \,\, \sum _{\begin{array}{c} n \in {\mathbb {Z}}^d \\ |n| \le N \end{array}} |c_D| \textrm{m}(D)^{-1/2} \textrm{m}\bigl (D \cap A^j ([0,1]^d + \ell + n)\bigr ) \\&= \sum _{\begin{array}{c} n \in {\mathbb {Z}}^d \\ |n| \le N \end{array}} \frac{1}{|\det A|^{j}} \sum _{\begin{array}{c} D \in {\mathcal {D}}_A \\ \textrm{scale}(D) \le j \end{array}} |c_D| \textrm{m}(D)^{-1/2} \textrm{m}\bigl (D \cap A^j ([0,1]^d + \ell + n)\bigr ). \end{aligned}$$

Note that $j = \textrm{scale}(A^j([0,1]^d + \ell + n)) = \textrm{scale} (D')$. Therefore, taking the supremum over all $D' = A^j ([0,1]^d + \ell )$ for $j \in {\mathbb {Z}}$ and $\ell \in {\mathbb {Z}}^d$ gives that

$$\begin{aligned} \sup _{D' \in {\mathcal {D}}_A} T_{D'}&\le \sum _{\begin{array}{c} n \in {\mathbb {Z}}^d \\ |n| \le N \end{array}} \sup _{j \in {\mathbb {Z}}, \ell \in {\mathbb {Z}}^d} \frac{1}{|\det A|^j} \sum _{\begin{array}{c} D \in {\mathcal {D}}_A \\ \textrm{scale}(D) \le j \end{array}} |c_D| \textrm{m}(D)^{-1/2} \textrm{m}\bigl (D \cap A^j ([0,1]^d + \ell + n)\bigr ) \\&\lesssim _{d,N} \sup _{D' \in {\mathcal {D}}_A} \frac{1}{\textrm{m}(D')} \sum _{\begin{array}{c} D \in {\mathcal {D}}_A \\ \textrm{scale}(D) \le \textrm{scale}(D') \end{array}} |c_D| m(D)^{-1/2} \textrm{m}(D \cap D'), \end{aligned}$$

which completes the proof. $\square$

For obtaining the actual dual characterization of the spaces $\dot{\textbf{f}}^0_{1, \infty } (A)$ and $\dot{\textbf{f}}^0_{\infty , 1}(A)$, the following lemma will be used. It is [17, Proposition 1.4] applied to the special case of dilated cubes; see also [25, Theorem 4] for the case of isotropic dilations.

### Lemma B.3

([17]) Let $a = (a_D)_{D \in {\mathcal {D}}_A}$ be a fixed but arbitrary sequence of non-negative reals. Then for every $C > 0$, the following assertions are equivalent:

(i) The sequence $a = (a_D)_{D \in {\mathcal {D}}_A}$ is a *C*-Carleson sequence, i.e.,
  B.7 $$\begin{aligned} \sum _{D \in {\mathcal {D}}'_A} a_D \le C \textrm{m}\bigg ( \bigcup _{D \in {\mathcal {D}}'_A} D \bigg ) \end{aligned}$$
  for every subcollection ${\mathcal {D}}'_A$ of the dilated cubes ${\mathcal {D}}_A$.
(ii) For every sequence $b = (b_D)_{D \in {\mathcal {D}}_A}$ of non-negative reals, the estimate
  $$\begin{aligned} \sum _{D \in {\mathcal {D}}_A} a_D b_D \le C \int _{{\mathbb {R}}^d} \sup _{D \in {\mathcal {D}}_A} b_D \mathbbm {1}_D (x) \; dx \end{aligned}$$
  holds.

The significance of a Carleson sequence (B.7) for the purpose of the present paper is that it characterizes membership of $\dot{\textbf{f}}_{\infty , 1}^0 (A)$. Although this fact is well-known for isotropic dilations (cf. [17, 25]), the anisotropic version requires some additional arguments due to the fact that dilated cubes are not necessarily nested. The details are provided in the next lemma.

### Lemma B.4

Let $A \in \textrm{GL}(d, {\mathbb {R}})$ be expansive and let $(c_D)_{D \in {\mathcal {D}}_A}$ be a complex-valued sequence. Then $c \in \dot{\textbf{f}}_{\infty , 1}^0(A)$ if, and only if, there exists $C > 0$ such that

B.8 $$\begin{aligned} \sum _{D \in {\mathcal {D}}_A'} |c_D| \textrm{m}(D)^{1/2} \le C \textrm{m}\bigg ( \bigcup _{D \in {\mathcal {D}}'_A} D \bigg ) \end{aligned}$$

for every subcollection ${\mathcal {D}}'_A \subseteq {\mathcal {D}}_A$. Moreover,

B.9 $$\begin{aligned} \Vert c \Vert _{\dot{\textbf{f}}^0_{\infty , 1}(A)} \asymp \inf \bigg \{ C >0 : \sum _{D \in {\mathcal {D}}_A'} |c_D| \textrm{m}(D)^{1/2} \le C \textrm{m}\bigg ( \bigcup _{D \in {\mathcal {D}}'_A} D \bigg ) \text { for all } {\mathcal {D}}'_A \subseteq {\mathcal {D}}_A \bigg \}, \end{aligned}$$

with implicit constant independent of *c*.

### Proof

First, it will be shown that if $(c_D)_{D \in {\mathcal {D}}_A}$ satisfies (B.8), then $(c_D)_{D \in {\mathcal {D}}_A} \in \dot{\textbf{f}}_{\infty , 1}^0(A)$. For this, let $D' \in {\mathcal {D}}_A$ be arbitrary. Then for any $C > 0$ satisfying (B.8), we have, by Lemma B.1,

$$\begin{aligned} \sum _{D \in {\mathcal {T}} (D')} |c_D| \textrm{m}(D)^{1/2} \le C \textrm{m}\bigg (\bigcup _{D \in {\mathcal {T}}(D')} D \bigg ) \le C \textrm{m}\bigg ( \bigcup _{\begin{array}{c} n \in {\mathbb {Z}}^d \\ |n| \le N \end{array}} D' + A^{\textrm{scale}(D')}n \bigg ) \lesssim C \textrm{m}(D'), \end{aligned}$$

with implicit constant independent of $D'$. Hence,

$$\begin{aligned} \frac{1}{\textrm{m}(D')} \sum _{D \in {\mathcal {T}}(D')} |c_D| \textrm{m}(D)^{1/2} \lesssim C, \end{aligned}$$

which yields $\Vert c \Vert _{\dot{\textbf{f}}_{\infty , 1}^0(A)} \lesssim C$ by Lemma B.2. This also implies $\lesssim$ in Eq. (B.9).

Conversely, let ${\mathcal {D}}_A' \subseteq {\mathcal {D}}_A$ be any subcollection. Note first that if, for all $N \in {\mathbb {N}}$, there exists some $D'\in {\mathcal {D}}_A'$ with $\textrm{scale}(D') > N$, then

$$\begin{aligned} \textrm{m}\bigg ( \bigcup _{D \in {\mathcal {D}}_A'} D \bigg ) \ge \textrm{m}(D') = |\det A|^{\textrm{scale}(D')} > |\det A|^N. \end{aligned}$$

Hence, $\textrm{m}\big ( \bigcup _{D \in {\mathcal {D}}_A'} D \big ) = \infty$ and (B.8) is trivially satisfied. Therefore, suppose throughout the remainder of the proof that there exists $N \in {\mathbb {N}}$ such that $\textrm{scale}(D) \le N$ for all $D \in {\mathcal {D}}_A'$. Set $j_1:= \max \{ \textrm{scale}(D): D \in {\mathcal {D}}_A' \} \le N$, and define

$$\begin{aligned} {\mathcal {D}}_1'':= \{ D \in {\mathcal {D}}_A': \textrm{scale}(D) = j_1 \}. \end{aligned}$$

Furthermore, set $({\mathcal {D}}_{1}'')^c:= \{ D \in {\mathcal {D}}_A': D \notin {\mathcal {T}}(D') \; \text {for any} \; D' \in {\mathcal {D}}_1'' \}.$ Observe that the elements of ${\mathcal {D}}_1''$ are pairwise disjoint up to measure zero. Moreover, by construction, the unions $\bigcup _{D' \in {\mathcal {D}}_1''} D'$ and $\bigcup _{D \in ({\mathcal {D}}_1'')^c} D$ are disjoint up measure zero and ${\mathcal {D}}_A' \subseteq \bigcup _{D \in {\mathcal {D}}''_1} {\mathcal {T}} (D) \cup ({\mathcal {D}}_{1}'')^c$.

For $\ell \ge 2$, we define ${\mathcal {D}}''_\ell$ inductively as follows: Set $j_\ell := \max \{ \textrm{scale}(D): D \in ({\mathcal {D}}_{\ell -1}'')^c \}$,

$$\begin{aligned} {\mathcal {D}}_{\ell } '':= \{ D \in ({\mathcal {D}}_{\ell -1}'')^c: \textrm{scale}(D) = j_\ell \}, \end{aligned}$$

and $({\mathcal {D}}_{\ell }'')^c:= \{D \in ({\mathcal {D}}_{\ell -1}'')^c:D \notin {\mathcal {T}}(D') \; \text {for any} \; D' \in {\mathcal {D}}_{\ell }''\}.$ Then, by construction, the dilated cubes in ${\mathcal {D}}_A'': = \bigcup _{\ell = 1}^{\infty } {\mathcal {D}}''_\ell$ are pairwise disjoint up to measure zero and

$$\begin{aligned} {\mathcal {D}}_A' \subseteq \bigcup _{\ell = 1}^{\infty } \bigcup _{D \in {\mathcal {D}}''_\ell } {\mathcal {T}} (D) = \bigcup _{D \in {\mathcal {D}}_A''} {\mathcal {T}} (D). \end{aligned}$$

Based on this construction, a direct calculation using Lemma B.2 yields

$$\begin{aligned} \sum _{D \in {\mathcal {D}}_A'} |c_D| \textrm{m}(D)^{1/2}&\le \sum _{D' \in {\mathcal {D}}_A''} \sum _{D \in {\mathcal {T}}(D')} |c_D| \textrm{m}(D)^{1/2} \lesssim \Vert c \Vert _{\dot{\textbf{f}}^0_{\infty , 1}(A)} \sum _{D' \in {\mathcal {D}}_A''} \textrm{m}(D') \\&= \Vert c \Vert _{\dot{\textbf{f}}^0_{\infty , 1}(A)} \, \textrm{m}\bigg ( \bigcup _{D' \in {\mathcal {D}}_A''} D'\bigg ) \le \Vert c \Vert _{\dot{\textbf{f}}^0_{\infty , 1}(A)} \, \textrm{m}\bigg (\bigcup _{D \in {\mathcal {D}}_A'} D \bigg ), \end{aligned}$$

where the last inequality used that ${\mathcal {D}}_A'' \subseteq {\mathcal {D}}_A'$. Hence $\Vert c \Vert _{\dot{\textbf{f}}^0_{\infty , 1}(A)}$ satisfies Eq. (B.8), which also implies the inequality $\gtrsim$ in (B.9). $\square$

A combination of Lemmata B.3 and B.4 yields the following dual characterization.

### Corollary B.5

Let $A \in \textrm{GL}(d, {\mathbb {R}})$ be expansive. Then, for all $c \in \dot{\textbf{f}}^0_{1, \infty } (A)$ and $c' \in \dot{\textbf{f}}^0_{\infty , 1} (A)$,

B.10 $$\begin{aligned} | \langle c, c' \rangle | := \bigg | \sum _{D \in {\mathcal {D}}_A} c_{D} \overline{c'_{D}} \bigg | \lesssim \Vert c \Vert _{\dot{\textbf{f}}^0_{1, \infty } (A)} \Vert c' \Vert _{\dot{\textbf{f}}^0_{\infty , 1} (A)} . \end{aligned}$$

Moreover, it holds that

B.11 $$\begin{aligned} \Vert c \Vert _{\dot{\textbf{f}}^0_{1, \infty } (A)} \asymp \sup \big \{ |\langle c, c' \rangle | \; : \; c' \in \dot{\textbf{f}}^0_{\infty , 1}(A), \;\; \Vert c' \Vert _{\dot{\textbf{f}}^0_{ \infty ,1}(A)} \le 1 \big \}. \end{aligned}$$

### Proof

For $c' \in \dot{\textbf{f}}^0_{ \infty , 1} (A)$ and $c \in \dot{\textbf{f}}^0_{1, \infty } (A)$, define sequences by $a_D:= |c'_D| \textrm{m}(D)^{1/2}$ and $b_D:= |c_D| \textrm{m}(D)^{-1/2}$ for $D \in {\mathcal {D}}_A$. Then, by Lemma B.4, we see that $(a_D)_{D \in {\mathcal {D}}_A}$ is a *C*-Carleson sequence, where $C \lesssim \Vert c' \Vert _{\dot{\textbf{f}}^0_{\infty , 1} (A)}$. By Lemma B.3, this implies

$$\begin{aligned} \sum _{D \in {\mathcal {D}}_A} |c_D c'_D| = \sum _{D \in {\mathcal {D}}_A} a_D b_D \le C \int _{{\mathbb {R}}^d} \sup _{D \in {\mathcal {D}}_A} b_D \mathbbm {1}_D (x) \; dx \lesssim \Vert c \Vert _{\dot{\textbf{f}}^0_{1, \infty } (A)} \Vert c' \Vert _{\dot{\textbf{f}}^0_{\infty , 1} (A)}, \end{aligned}$$

showing Eq. (B.10).

To obtain the dual characterization (B.11), we follow [28, Section 69] and define the associate norms of $\Vert \cdot \Vert _{\dot{\textbf{f}}^0_{1, \infty }}$ by $\Vert \cdot \Vert ^{(0)}:= \Vert \cdot \Vert _{\dot{\textbf{f}}^0_{1, \infty }}$ and

$$\begin{aligned} \Vert c \Vert ^{(n)}:= \sup \bigg \{ \sum _{D \in {\mathcal {D}}'_A} |c_D c'_D| \;: \; \Vert c' \Vert ^{(n-1)} \le 1 \bigg \} = \sup \{ | \langle c, c' \rangle | \;: \; \Vert c' \Vert ^{(n-1)} \le 1 \}, \quad n \ge 1, \end{aligned}$$

where the equality can be shown using the solidity of the associate norms and choosing sequences $c'$ with appropriate (complex) signs; see also [28, Section 69, Theorem 1] for details. In the following, we consider $\Vert \cdot \Vert ^{(1)}$ and $\Vert \cdot \Vert ^{(2)}$ in more detail. Starting with $\Vert \cdot \Vert ^{(1)}$, we interpret the supremum as an infimum over all upper bounds. Then the characterizations of Lemma B.3 and B.4 give

$$\begin{aligned} \Vert c \Vert ^{(1)}&= \sup \bigg \{ \sum _{D \in {\mathcal {D}}'_A} |c_D c'_D| : \Vert c' \Vert _{\dot{\textbf{f}}^0_{1,\infty }} \le 1 \bigg \} \\&= \inf \bigg \{ C> 0 : \sum _{D \in {\mathcal {D}}'_A} |c_D c'_D| \le C \Vert c' \Vert _{\dot{\textbf{f}}^0_{1,\infty }} \text { for all } c' \in \dot{\textbf{f}}^0_{1,\infty }(A) \bigg \} \\&= \inf \bigg \{ C > 0 : \sum _{D \in {\mathcal {D}}_A'} |c_D| \textrm{m}(D)^{1/2} \le C \textrm{m}\bigg ( \bigcup _{D \in {\mathcal {D}}'_A} D \bigg ) \text { for all } {\mathcal {D}}'_A \subseteq {\mathcal {D}}_A \bigg \} \\&\asymp \Vert c \Vert _{\dot{\textbf{f}}^0_{\infty , 1}(A)}. \end{aligned}$$

The Lorentz-Luxemburg duality theorem for normed Köthe spaces (see, e.g., [28, Section 71, Theorem 1]) states that $\Vert \cdot \Vert _{\dot{\textbf{f}}^0_{1, \infty }} = \Vert \cdot \Vert ^{(2)}$ provided $\Vert \cdot \Vert _{\dot{\textbf{f}}^0_{1, \infty }}$ satisfies the Fatou property. Since the latter is a straightforward consequence of Fatou’s lemma and [28, Section 65, Theorem 3]), we obtain

$$\begin{aligned} \Vert c \Vert _{\dot{\textbf{f}}^0_{1, \infty } (A)} = \Vert c \Vert ^{(2)} = \sup \big \{ |\langle c, c' \rangle | \;: \; \Vert c' \Vert ^{(1)} \le 1 \big \} \asymp \sup \big \{ |\langle c, c' \rangle | \;: \; \Vert c' \Vert _{\dot{\textbf{f}}^0_{\infty , 1}(A)} \le 1 \big \} \end{aligned}$$

for arbitrary $c \in \dot{\textbf{f}}^0_{1, \infty } (A)$. This completes the proof. $\square$

The final result of this section is the desired dual norm characterization of $\dot{\textbf{F}}^{0}_{1,\infty }(A)$.

### Proposition B.6

Let $A \in \textrm{GL}(d, {\mathbb {R}})$ be expansive. Then, for all $g \in {\mathcal {S}}_0({\mathbb {R}}^d)$,

$$\begin{aligned} \Vert g \Vert _{\dot{\textbf{F}}^{0}_{1,\infty }(A)} \asymp \sup _{\begin{array}{c} f \in \dot{\textbf{F}}^0_{\infty , 1} (A) \\ \Vert f \Vert _{\dot{\textbf{F}}^0_{\infty , 1} (A)} \le 1 \end{array} } |\langle f, g\rangle |. \end{aligned}$$

### Proof

By [4, Theorem 3.12], there exists a function $\psi \in {\mathcal {S}} ({\mathbb {R}}^d)$ with compact Fourier support such that the operator $\mathscr {C}_{\psi } f = (\langle f, \psi _D \rangle )_{D \in {\mathcal {D}}}$ is bounded from $\dot{\textbf{F}}^{0}_{p,q}(A)$ into $\dot{\textbf{f}}^{0}_{p,q}(A)$ and furthermore the operator $\mathscr {D}_{\psi } c = \sum _{D \in {\mathcal {D}}} c_D \psi _D$ is bounded from $\dot{\textbf{f}}^{0}_{p,q}(A)$ into $\dot{\textbf{F}}^{0}_{p,q}(A)$ for all $p,q \in (0, \infty ]$. Moreover, their composition $\mathscr {D}_{\psi } \circ \mathscr {C}_{\psi }$ is the identity on $\dot{\textbf{F}}^{0}_{p,q}(A)$. Here, for $D = A^{j}([0,1]^d + k)$, the function $\psi _D$ is defined as $\psi _D (x) = |\det A|^{-j/2} \psi (A^{-j} x - k)$; see [4, Equation (2.9)].

Next, [6, Lemma 2.8] implies for all $f \in {\dot{\textbf{F}}^0_{\infty , 1} (A)} \subseteq {\mathcal {S}}' / {\mathcal {P}} \cong {\mathcal {S}}_0'({\mathbb {R}}^d)$ and $g \in {\mathcal {S}}_0 ({\mathbb {R}}^d) \subseteq {\dot{\textbf{F}}^0_{1, \infty } (A)}$ that

$$\begin{aligned} \langle f, g \rangle = \sum _{D \in {\mathcal {D}}_A} \langle f, \psi _{D} \rangle \langle \psi _{D}, g \rangle = \sum _{D \in {\mathcal {D}}_A} \langle f, \psi _{D} \rangle \overline{\langle g, \psi _{D} \rangle } = \big \langle \mathscr {C}_{\psi } f, \mathscr {C}_{\psi } g \rangle . \end{aligned}$$

Combining both facts with the estimate (B.10), it follows that

$$\begin{aligned} |\langle f, g \rangle | = | \langle \mathscr {C}_{\psi } f, \mathscr {C}_{\psi } g \rangle | \lesssim \Vert \mathscr {C}_{\psi } f \Vert _{\dot{\textbf{f}}^0_{\infty , 1} (A)} \Vert \mathscr {C}_{\psi } g \Vert _{\dot{\textbf{f}}^0_{1, \infty } (A)} \lesssim \Vert f \Vert _{\dot{\textbf{F}}^0_{\infty , 1} (A)} \Vert g \Vert _{\dot{\textbf{F}}^0_{1, \infty } (A)}. \end{aligned}$$

For the reverse inequality, first note that since $\mathscr {D}_{\psi } \circ \mathscr {C}_{\psi }$ is the identity on $\dot{\textbf{F}}^0_{1, \infty } (A)$, and since these operators are bounded, we have

$$\begin{aligned} \Vert g \Vert _{\dot{\textbf{F}}^0_{1, \infty } (A)} = \Vert \mathscr {D}_{\psi } \mathscr {C}_{\psi } g \Vert _{\dot{\textbf{F}}^0_{1, \infty } (A)} \lesssim \Vert \mathscr {C}_{\psi } g \Vert _{\dot{\textbf{f}}^0_{1, \infty } (A)} \lesssim \Vert g \Vert _{\dot{\textbf{F}}^0_{1, \infty } (A)} \end{aligned}$$

and thus $\Vert \mathscr {C}_{\psi } g \Vert _{\dot{\textbf{f}}^0_{1, \infty } (A)} \asymp \Vert g \Vert _{\dot{\textbf{F}}^0_{1, \infty } (A)}$. Next, note that by Corollary B.5, there exists a sequence $c' \in \dot{\textbf{f}}^0_{ \infty , 1} (A)$ with $\Vert c' \Vert _{\dot{\textbf{f}}^0_{ \infty , 1} (A)} = 1$ such that

$$\begin{aligned} | \langle \mathscr {C}_{\psi } g, c' \rangle | \asymp \Vert \mathscr {C}_{\psi } g \Vert _{\dot{\textbf{f}}^0_{1, \infty } (A)}. \end{aligned}$$

Now, setting $f:= \mathscr {D}_{\psi } c' \in \dot{\textbf{F}}^0_{\infty , 1} (A)$, note that

$$\begin{aligned} \langle f, g \rangle = \langle \mathscr {D}_{\psi } c', g \rangle = \sum _{D \in {\mathcal {D}}_A} c'_D \, \langle \psi _D, g \rangle = \sum _{D \in {\mathcal {D}}_A} c'_D \, \overline{(\mathscr {C}_{\psi } g)_D} = \langle c', \mathscr {C}_{\psi } g \rangle . \end{aligned}$$

It follows that

$$\begin{aligned} | \langle f, g \rangle | = |\langle c', \mathscr {C}_{\psi } g \rangle | = | \langle \mathscr {C}_{\psi } g, c' \rangle | \asymp \Vert \mathscr {C}_{\psi } g \Vert _{\dot{\textbf{f}}^0_{1, \infty } (A)} \asymp \Vert g \Vert _{\dot{\textbf{F}}^0_{1, \infty } (A)}. \end{aligned}$$

Since $\mathscr {D}_{\psi } :\dot{\textbf{f}}^0_{\infty , 1} (A) \rightarrow \dot{\textbf{F}}^0_{\infty ,1} (A)$ is bounded, we have $\Vert f\Vert _{\dot{\textbf{F}}^0_{\infty ,1} (A)} \le C \Vert c'\Vert _{\dot{\textbf{f}}^0_{\infty ,1} (A)} \le C$. Hence, normalizing *f* by $C > 0$ if necessary yields that

$$\begin{aligned} \Vert g \Vert _{\dot{\textbf{F}}^0_{1, \infty } (A)} \lesssim \sup _{\begin{array}{c} f \in \dot{\textbf{F}}^0_{\infty , 1} (A) \\ \Vert f \Vert _{\dot{\textbf{F}}^0_{\infty , 1}(A)} \le 1 \end{array}} |\langle f, g\rangle |, \end{aligned}$$

which finishes the proof. $\square$
